# A multi-epitope approach for development of a universal vaccine against leptospirosis

**DOI:** 10.3389/fimmu.2026.1774717

**Published:** 2026-03-20

**Authors:** Mohd Abdullah, Mohammad Kadivella, Pallavi Vyas, Jusail C. P., Bothammal Palanisamy, Sarwar Azam, Syed M. Faisal

**Affiliations:** 1 Laboratory of Vaccine Immunology, National Institute of Animal Biotechnology, Hyderabad, India; 2 Laboratory of Genomics and Computational Biology, National Institute of Animal Biotechnology, Hyderabad, India; 3 Regional Centre for Biotechnology, Faridabad, India

**Keywords:** *Leptospira*, leptospirosis, multi-epitope-vaccine, next generation vaccine, reverse vaccinology

## Abstract

**Introduction:**

*Leptospira*, a zoonotic pathogen, poses a significant public health threat, causing morbidity and mortality in both humans and animals. Although several bacterin and recombinant vaccines targeting specific serovars have been developed, most provide short-term, serovar-restricted protection and do not consistently induce sterilizing immunity or broad cross-serovar coverage.

**Materials:**

To address this challenge, we employed comparative pan-genomic analysis and reverse vaccinology to identify conserved potential vaccine candidates (PVCs) and designed a multi-epitope vaccine (MEV) incorporating highly immunogenic B- and T-cell epitopes. Unlike previous studies largely limited to computational prediction or a small number of known antigens, our strategy integrates soft-core genome-wide antigen prioritization with structural validation and experimental evaluation of innate and adaptive immune responses. Selected epitopes were linked with appropriate spacers and fused to a TLR4 agonist (APPHALS) at the N-terminus to enhance immune activation.

**Results and discussion:**

*In silico* analyses confirmed the stability and immunogenic potential of the MEV construct. The purified recombinant MEV reacted strongly with hyperimmune and clinical sera. Anti-MEV antibodies agglutinated multiple pathogenic *Leptospira* serovars and inhibited bacterial growth *in vitro*. MEV stimulation induced macrophage activation, evidenced by increased proinflammatory cytokine production and upregulation of co-stimulatory molecules. Immunization in mice elicited robust humoral and T-cell responses. Collectively, these findings position MEV as a rationally designed next-generation vaccine candidate with potential for broad cross-protective immunity against leptospirosis.

## Highlights

- A novel multi-epitope vaccine (MEV) against *Leptospira* was developed using comparative genomics and reverse vaccinology, incorporating B- and T-cell epitopes fused with a TLR4 agonist to enhance immune response. The MEV showed strong immunogenicity, cross-reactive antibody generation, and macrophage activation *in vitro*. It also elicited robust humoral and T cell responses in mice without the need for an adjuvant, demonstrating its potential as a cross-protective vaccine candidate.

## 1 Introduction

Leptospirosis is a disregarded zoonotic disease produced by pathogenic *Leptospira* species, with worldwide frequency (1, 2). Human infection transpires through direct contact with diseased animals or indirectly through polluted water as well as soil contaminated by infected animal urine. Leptospirosis is expected to account for more than one million cases per year (3–6). Clinical manifestations in humans vary from mild, nonspecific symptoms (e.g., fever, headache and muscle ache) to severe conditions such as Weil’s syndrome or severe pulmonary hemorrhage syndrome (SPHS), which may exhibit mortality rates surpassing 70% (7). In animals, leptospirosis is chiefly linked to reproductive failure and output losses, leading to considerable economic burdens in the cattle sector. Current available veterinary vaccines, such as SPIROLET, Vax-Spiral, and Nobivac L4, are based on inactivated whole-cell bacterins (8–10). However, these vaccines provide only short-term, serovar-specific immunity due to their reliance on lipopolysaccharides (LPS) and do not induce sterilizing immunity. Additionally, their toxicity limits their use in humans (11–14). This underscores the immediate necessity for the development of an effective, non-toxic, and widely protective vaccination that can elicit long-lasting cross-protective immunity among host species.

Numerous surface and outer membrane proteins of *Leptospira* were investigated as possible subunit vaccine candidates, exhibiting differing levels of protection in animal models (15, 16). *Leptospira* immunoglobulin-like protein A (LigA), especially its C-terminal or variable region (LAV), has demonstrated potential in many investigations (17, 18).

However, these proteins have conferred moderate to high levels of protection (40–83% survival), yet failed to achieve sterilizing immunity or consistent cross-protection (19). Given the diverse repertoire of surface proteins involved in *Leptospira* pathogenesis, single-antigen-based vaccines may be insufficient to confer comprehensive protection. Consequently, multi-component vaccine formulations incorporating multiple protective antigens have been developed to improve immunogenicity and generate more robust immune responses compared to single-antigen vaccines (20, 21) Although these formulations have shown improved protective efficacy, achieving sterilizing immunity and broad cross-serovar protection remains a major challenge in leptospiral vaccine development.

An example of a modern vaccine construction method is the multi-epitope vaccine (MEV), which combines several protein epitopes into one subunit vaccination by employing linker sequences (20–22). MEVs can stimulate more robust T and B cell immune responses than full length proteins, as entire proteins are not always necessary to elicit immunity (23–26). This approach has shown promise, with several MEVs currently undergoing clinical trials (27, 28). While several multi-epitope or chimeric vaccine constructs targeting *Leptospira* have been proposed in recent reverse vaccinology studies, most have been evaluated primarily through in silico analyses, with limited *in vitro* and *in vivo* validation (14, 29, 30). Several contemporary immunoinformatics-driven vaccine design studies have described multi-epitope and chimeric constructs derived from selected outer membrane proteins of *Leptospira*, including LruC and members of the LipL family (31–33). While these reports demonstrate promising immunogenic profiles, their antigen selection strategies are largely centered on predefined protein candidates rather than systematic pan-genome prioritization of conserved virulence-associated targets across pathogenic *Leptospira* species. Moreover, these vaccines have been constructed using epitopes from a few known protective antigens, rather than incorporating conserved and highly immunogenic antigens across diverse pathogenic *Leptospira* serovars. As a result, few of those tested have demonstrated only partial protection without sterilizing immunity in hamster models (34–36). To address these limitations, a MEV incorporating the best epitopes from highly immunogenic and conserved antigens across diverse pathogenic *Leptospira* serovars is required as a foundational step toward developing a universal leptospirosis vaccine.

A soft-core genome comprising 2,408 genes was discovered in our recent study by doing comparative genomics of all sequenced pathogenic *Leptospira* genomes comprising these 1,478 genes were detected in all species, while the remaining genes were found in at least 95% of species. Using a reverse vaccinology approach, we identified potential vaccine candidates (PVCs), which were subsequently utilized for the *in silico* design and characterization of an MEV. The purified recombinant MEV was then tested for immune reactivity and innate immune activation *in vitro*, followed by an evaluation of its immunogenicity in a mouse model.

## 2 Materials and methods

### 2.1 Cell and bacterial culture and reagents

RAW 264.7 murine macrophage cells (ATCC^®^ TIB-71™, American Type Culture Collection, Manassas, VA, USA) were cultured in Dulbecco’s Modified Eagle Medium (DMEM; Cat# D6429, Sigma-Aldrich, St. Louis, MO, USA) supplemented with 10% fetal bovine serum (FBS; Gibco™, Thermo Fisher Scientific, Waltham, MA, USA), and 1X Antibiotic-Antimycotic (Gibco™, Thermo Fisher Scientific, Waltham, MA, USA) at 37°C in a humidified incubator with 5% CO_2_. Pathogenic *Leptospira* strains were obtained from ICAR-NIVEDI (Bengaluru, India) and ICMR-NIE (Chennai, India) and maintained in Ellinghausen–McCullough–Johnson–Harris (EMJH) medium supplemented with 10% enrichment (Difco™, BD Biosciences, Franklin Lakes, NJ, USA) at 28°C. Cytokine sandwich ELISA kits for murine IL-6, IL-4, TNF-α, and IFN-γ were purchased from R&D Systems, Minneapolis, MN, USA. Flow cytometry antibodies were obtained from BD Biosciences, San Jose, CA, USA, unless otherwise specified.

### 2.2 Animals

Female BALB/c mice, aged 4–6 weeks, were obtained from the Animal Resource and Experimental Facility at NIAB in Hyderabad, India. The animals were kept in standard pathogen-free conditions, with unrestricted access to food and water. All animal experiments were carried out in full compliance with the guidelines set forth by the Committee for the Purpose of Control and Supervision of Experiments on Animals (CPCSEA) and the Institutional Animal Ethics Committee (IAEC) under protocol number (IAEC/NIAB/2023/54/SF). All relevant ethical regulations for animal use have been thoroughly compiled.

### 2.3 Reverse vaccinology approach for identification of potential vaccine candidates from *Leptospira* genome

Reverse vaccinology approach was utilized to identify the potential vaccine candidates against leptospirosis. To do so we predicted softcore proteome of pathogenic *Leptospira* strains, as reported in our previous study (37). A multi-step *in silico* pipeline was applied to identify immunogenic proteins suitable for vaccine development. Initially, PSORTb v3.0.2 (https://www.psort.org/psortb/) was used to predict the sub cellular localization, and proteins localized to the outer membrane (OM) or extracellular (EC) space were shortlisted as potential surface-exposed antigens (38). Transmembrane helices were predicted using TMHMM Server v2.0 (www.cbs.dtu.dk/services/TMHMM) and proteins with multiple transmembrane regions were excluded due to their complex structure and expression challenges (39). Next, adhesins and adhesin-like proteins were predicted from the shortlisted set using SPAAN software (40). To eliminate candidates with potential cross-reactivity to human proteins, sequence similarity analysis was performed using previously reported thresholds. Protein sequences exhibiting more than 35% identity across an 80 amino acid window or any uninterrupted sequence of 9 or more identical residues, were excluded from consideration (41–43). The complete human proteome, obtained from NCBI (https://www.ncbi.nlm.nih.gov/genome/guide=/human/), was used for this homology analysis, which was conducted using custom in-house Perl scripts. Candidate adhesins were further screened for antigenicity using VaxiJen v2.0 with a threshold score of 0.5 (44). Proteins predicted to be allergenic, identified using the AllergenOnline database (http://www.allergenonline.org/databasefasta.shtml) with >35% identity over an 80 amino acid sliding window were also excluded (45). To prioritize proteins with protective potential, VaxiGen-ML (http://www.violinet.org/vaxign/vaxign-ml/index.php) was used to assess protective antigenicity, and proteins having a protegenicity score ≥ 90 were further selected for downstream analysis (46). For B-cell epitope prediction, BCPREDS software was used, applying both the Amino Acid Pair (AAP) antigenicity method and the other subsequence kernel approach (http://ailab-projects2.ist.psu.edu/bcpred/predict.html) (47). Epitopes with length ≥20 amino acids and specificity >80% were considered, and overlapping predictions from both methods were designated as consensus B-cell epitopes (48). Additionally, BepiPred v2.0 from the IEDB was used to validate B-cell epitope predictions using a 20-residue threshold (49).

T-cell epitopes were predicted using the standard settings of IEDB MHC-I (v2.23) and MHC-II (v2.22) prediction tools (50). We ensured broad global coverage by using a reference set of MHC-I and MHC-II alleles. For MHC-I, peptides with a consensus percentile rank <0.1% were selected, while MHC-II strong binders were filtered using a <1% threshold. Epitopes binding to more than four HLA alleles were classified as promiscuous epitopes (51, 52). The immunogenic potential of MHC-I-restricted peptides was assessed using the IEDB “T cell class I pMHC immunogenicity predictor” (53), and cumulative immunogenicity scores were calculated based on all predicted 9-mer epitopes (46).

The population coverage of selected MHC class I and class II epitopes was evaluated using the IEDB Population Coverage Tool v1.0.1 (54). The analysis was performed using reference HLA allele frequency datasets available within the IEDB database. Population coverage was calculated individually for India, Turkey, and Saudi Arabia, and comparatively for broader geographic regions including South Asia, Southwest Asia (Middle East), and the global population. The combined MHC-I and MHC-II restricted epitope coverage was determined to estimate cumulative population representation. The immunogenicity and immune response characteristics of the chosen vaccination candidates were simulated via the C-ImmSim server with default parameters, which simulates host immune responses through machine learning and position-specific scoring matrices (55).

### 2.4 *In silico* engineering of a novel multi-epitope vaccine: structure, TLR4 DOCKING, and molecular dynamics simulations

Promiscuous MHC-I, MHC-II and high-confidence B-cell epitopes from the selected vaccine candidates were clustered using the Epitope Cluster Analysis Tool (http://tools.iedb.org/cluster/) (56), with grouping based on 70% sequence identity. The multi-epitope vaccine design incorporated at least one B-cell, MHC-I and II epitope from each candidate, prioritizing epitopes with high antigenicity, immunogenicity, and promiscuity, and preferring those overlapping across all classes (57). The hydropathy index of each epitope was calculated using GRAVY analysis from ProtParam (https://web.expasy.org/protparam/) (58). Based on hydropathy values, epitopes were arranged and linked using “GGGGS” for B-cell, “GGGS” for MHC-I, and “GPGPG” for MHC-II epitopes. Additionally, an “EAAAK” linker was added to join the TLR-4 adjuvant “APPHALS” at the N-terminal of the MEV construct (59). Solubility was evaluated using SOLpro (60), whilst theoretical isoelectric point (pI), molecular weight (MW), aliphatic index, half-life, GRAVY were calculated with ProtParam and Allergen Online. Allergenicity and anti-inflammatory characteristics were assessed using AIPpred (61), whereas antigenicity was predicted with VaxiJen v2.0 (44). The C-ImmSim server generated immune response profiles (55). The Chou and Fasman Secondary Structure Prediction (CFSSP) server (48) (http://www.biogem.org/tool/chou-fasman/index.php) analyzed the vaccine’s alpha-helices, beta-sheets, and turns, while RaptorX predicted solvent accessibility and generated the 3D structure (62). The structure was refined using ModRefiner (63) (https://zhanggroup.org/ModRefiner/), and its quality was assessed through Ramachandran plot analysis using Procheck (SAVES v6.0, https://saves.mbi.ucla.edu/) (64) and ProSA-web (https://prosa.services.came.sbg.ac.at/prosa.php) (65). The PatchDock web server (https://bioinfo3d.cs.tau.ac.il/PatchDock/) was employed to dock the MEV 3D structure with human TLR4 receptor (PDB ID: 4G8A) (66), followed by refinement with FireDock (https://pubmed.ncbi.nlm.nih.gov/17598144/) (67). Docked complexes were visualized using PDBSum and UCSF Chimera v1.15 (68). Molecular dynamics (MD) simulations were performed using GROMACS v2020.5 to assess the stability and energy minimization of the human TLR4-MEV complex with the lowest binding energy (69). The OPLS-AA force field (70) and TIP3P water model (71) were applied. Energy optimization used the steepest decline approach until the maximum force fell below 1000.0 kJ/mol/nm. The system was equilibrated under NPT and NVT ensembles for 100 ps each at 1 bar pressure and 300 K. A final production MD run of 100 ps with 500,000 steps was then conducted.

### 2.5 MEV synthesis and protein purification

Using the JCat server (http://www.prodoric.de/JCat), codon optimization was carried out to improve expression efficiency in *Escherichia coli* strain K12 (72). The optimization process followed these criteria: (i) avoiding termination of rho-independent transcription, (ii) preventing prokaryotic ribosome-binding sites, and (iii) eliminating restriction enzyme cleavage sites for EcoRI and HindIII. Following optimization, the sequence was inserted between the EcoRI and HindIII cleavage sites of the pUC57 plasmid. *In silico* cloning and visualization were performed using SnapGene software (https://www.snapgene.com). The MEV sequence was synthesized (GeneScript Biotech Corporation, China) and inserted between the EcoRI and HindIII cleavage sites of the pUC57 plasmid. The MEV gene was then subcloned into His-tagged pET28a (+) expression vector. The construct was verified by Sanger sequencing. Recombinant MEV, recombinant LigA (C-terminal region), and the leptospiral outer membrane lipoprotein LipL32 were expressed as His-tagged fusion proteins in *E. coli* BL21 (DE3) cells as described previously (73). Recombinant LigA was included as a well-characterized protective antigen and used as a comparative control in macrophage stimulation experiments (18, 74, 75) Briefly, overnight cultures were cultivated at 37°C in LB broth with 50 µg/ml kanamycin, then inoculated into fresh media and cultured to an OD_600_ of ~0.5. Protein expression was induced with 1 mM IPTG, at 18°C overnight. Cells were collected via centrifugation at 6000 rpm and resuspended in a buffer of 50 mM NaH_2_PO_4_, 300 mM NaCl, 20 mM imidazole, and 1% N-lauroylsarcosine sodium salt (pH 7.4) before sonication. The lysate was centrifuged to eliminate cellular debris, and the supernatant was utilized to purified protein using Ni-NTA affinity resin (Takara, Japan). Protein expression and purity were validated via SDS-PAGE. The quantity of protein was determined via the Bradford reagent (Takara, Japan). After SDS-PAGE, the purified MEV was transferred onto PVDF membranes (Millipore, Bedford, MA) and blocked with 5% non-fat milk in TBST (pH 7.4, 0.1% Tween-20). Anti-His antibody (Cell Signaling Technology, Beverly, MA) was used to incubate the membranes overnight at 4°C, then subsequently rinsed in TBST and incubated for one hour at room temperature using HRP-conjugated anti-mouse IgG secondary antibody (1:3000, CST, USA). Protein bands were detected utilizing Clarity™ Western ECL substrate (Bio-Rad Laboratories, Hercules, CA, USA) in the ChemiDoc System (Bio-Rad Laboratories, Hercules, CA, USA).

### 2.6 MEV reactivity and specificity

#### 2.6.1 Indirect ELISA

The antigenic reactivity of the MEV was assessed through an indirect ELISA using two different serum panels: (i) human sera, including those from individuals with confirmed leptospirosis by the microscopic agglutination test (MAT-positive), patients diagnosed with other febrile illnesses, and healthy volunteers, and (ii) hyperimmune sera obtained from mice experimentally infected with *Leptospira interrogans* serovar Pomona, collected 21 days after infection. The ELISA procedure followed a standard format. In brief, 96-well plates were coated overnight at 4°C with 100 µL of MEV solution (200 ng per well). The plates were then washed three times with PBS supplemented with 0.05% Tween-20 (PBST) and blocked with 300 µL of 1% BSA in PBS for 1 hour at room temperature (RT). Subsequently, 100 µL of each serum sample, diluted 1:100 in PBS, was added to the wells and incubated for 2 hours at RT. After washing three times with PBST, bound antibodies were detected with either HRP-conjugated anti-human IgG (1:6000; Southern Biotech, USA) or HRP-conjugated anti-mouse IgG (1:6000; Southern Biotech, USA), followed by a 2-hour incubation at RT. Plates were again washed three times before adding 100 µL of TMB substrate (MP Biomedicals, USA) and incubating for 20 minutes at RT in the dark. The reaction was terminated by adding 50 µL of 2 N sulfuric acid, and absorbance was read at 450 nm using a multimode microplate reader (PerkinElmer, USA).

#### 2.6.2 Microscopic agglutination test

The MAT was performed to determine whether antibody raised against the MEV protein could agglutinate various *Leptospira* serogroups, following established protocols (76). Briefly, mouse anti-MEV or hyperimmune sera (positive control) or healthy sera (negative control) were serially diluted two-fold in PBS, from 1:20 to 1:2560, in a microtiter plate. 25 µL of each dilution was added to a fresh plate, and an equal volume (25 µL) of live *Leptospira* spp. suspension (~1 × 10^8^ cells/mL; see **Supplementary Table 1**) was added and mixed gently. The plates were incubated for 2–4 hour at 30 °C, and agglutination was assessed under a dark-field microscope (Zeiss, Germany). Representative fields were imaged.

#### 2.6.3 Dot blot assay

A dot blot assay was carried out in accordance with a previously established procedure, with few modifications (77). The assay was used to evaluate MEV reactivity across different *Leptospira* serogroups. Heat-killed *Leptospira* antigens (HKL) were prepared from a panel of 12 *Leptospira* cultures (**Supplementary Table 1**). HKL samples (5 µL containing 3 µg protein) were dotted onto a nitrocellulose membrane and air-dried. The membrane was incubated with 5% (w/v) non-fat milk in TBST [Tris-buffered saline with 0.1% (v/v) Tween-20] for 1 hour at room temperature, followed by four washes with TBST. The membrane was subsequently treated with anti-MEV serum and pre-immune serum (diluted 1:200 in TBST) for one hour at room temperature and rinsed three times with TBST. Thereafter, the membrane was incubated with HRP-conjugated anti-mouse IgG (1:3000, CST, USA) for one hour at room temperature, followed by three washes with TBST. Peroxidase-positive signals were identified utilizing Clarity™ Western ECL substrate (Bio-Rad Laboratories, Hercules, CA, USA) in the ChemiDoc System (Bio-Rad Laboratories, Hercules, CA, USA). Band intensities were assessed via ImageJ software and represented in arbitrary units (AU).

### 2.7 Cell stimulation assay

RAW 264.7 macrophages (1 × 10^6^ cells per well) were seeded in complete DMEM and maintained at 37°C in a humidified atmosphere containing 5% CO_2_. Cells were exposed for 24 hours to different concentrations of MEV (0.25–2 µg/mL). Recombinant LigA (2 µg/mL) was included as a protein control to compare innate immune activation. Lipopolysaccharide (LPS; 500 ng/mL) served as a positive control, while untreated cells were used as a negative control. To control for endotoxin contamination, MEV preparations were incubated with Polymyxin B (10 µg per mg of protein; InvivoGen) at 37°C for 1 hour prior to stimulation. For evaluation of protein-specific responses, MEV was subjected to digestion with Proteinase K (5 µg per mg of protein; Sigma) at 65°C for 1 hour, followed by enzyme inactivation at 95°C for 5 minutes. After the stimulation period, cell-free supernatants were collected for cytokine measurement using sandwich ELISA, and the remaining cells were harvested for RNA extraction and subsequent RT-qPCR analysis of innate immune-related genes.

#### 2.7.1 Cytokine ELISA

Supernatants from RAW 264.7 cultures, harvested 24 hours following stimulation, were assessed for IL-6 and TNF-α production using commercially available sandwich ELISA kits (R&D Systems), following the protocols provided by the manufacturer. Optical density was recorded at 450 nm with background correction at 570 nm. Cytokine concentrations were determined by comparison with standard curves prepared using recombinant cytokine standards.

#### 2.7.2 Flow cytometry analysis

RAW 264.7 cells (1 × 10^6^/well) were stimulated for 24 hours at 37°C, 5% CO_2_ with LPS (500 ng/mL) or Polymyxin B treated MEV (2 µg/mL). Cells were washed with ice-cold PBS, blocked (PBS + 0.5% BSA + 2% FBS, 20 min, 4°C), and stained for 1 hour at 4°C in the dark with PE-anti-mouse I-A/I-E (MHC II; Cat# 557000), APC-anti-mouse CD80 (Cat# 560016), and PE-anti-mouse CD86 (Cat# 553692) (all BD Biosciences, USA). After PBS washes, cells were acquired on an LSR Fortessa (50,000 events/sample). Data were analyzed in FlowJo with appropriate unstained and single-stain controls.

#### 2.7.3 RT–qPCR analysis

After stimulation, RAW 264.7 cells were harvested, and toal RNA was extracted using the NucleoSpin RNA Kit (Cat# 740955.25, Takara) following the manufacturer’s guidelines. The quality and concentration of RNA were assessed with a NanoDrop 2000 spectrophotometer (Thermo Scientific). Complementary DNA (cDNA) was synthesized from purified RNA using the PrimeScript 1st Strand cDNA Synthesis Kit (Cat# 6110A, Takara, Japan). Quantitative PCR was performed in a total reaction volume of 10 µL containing 50 ng of cDNA, 10 µM of each gene-specific primer (listed in **Supplementary Table 2**), and iTaq Universal SYBR Green Supermix (Cat# 1725124, Bio-Rad) on a CFX Opus 96 Real-Time PCR System (Bio-Rad). Each sample was analyzed in triplicate. Relative expression levels were normalized against GAPDH and presented as log_2_ fold changes.

### 2.8 Testing the immunogenicity of MEV

#### 2.8.1 Immunization

BALB/c female mice (4–6 weeks old; five animals per group) were immunized subcutaneously with 10 µg of recombinant MEV formulated with or without alum adjuvant. The alum used was Alhydrogel^®^ 2% (aluminum hydroxide gel) at a 1:1 volume ratio with the antigen. A booster dose of 10 µg recombinant MEV, with or without alum, was administered on day 21 post-immunization. Blood samples were collected at three time points: pre-immunization (pre-bleed), day 21 (before booster), and day 28 (endpoint). On day 28, mice were euthanized following institutional ethical guidelines, and blood and spleen samples were collected for the analysis of antigen-specific antibody and T-cell responses.

#### 2.8.2 Evaluation of antibody response

Antibody responses in sera from immunized mice were quantified by indirect ELISA. In brief, 96-well microplates (Nunc, Denmark) were coated with MEV at a concentration of 200 ng per well in PBS and incubated overnight at 4°C. The plates were then washed three times with PBS containing 0.05% Tween-20 (PBST) and blocked with 1% BSA in PBS for 1 hour at room temperature. Following blocking, 100 µL of serially diluted serum samples (ranging from 1:100 to 1:100,000 in PBS containing 1% BSA) were added to each well and incubated for 2 hours at room temperature. After washing, wells were incubated with 100 µL of HRP-conjugated goat anti-mouse IgG, IgG1, IgG2a, IgG2b, or IgG2c (1:6000 dilution; SouthernBiotech) for 2 hours at room temperature. The plates were washed again, developed with 100 µL of TMB substrate (MP Biomedicals) for 20 minutes in the dark, and the reaction was stopped by adding 50 µL of 2 N H_2_SO_4_;. Absorbance values were recorded at 450 nm using a multimode plate reader (PerkinElmer).

#### 2.8.3 Evaluation of T-cell response

Spleens collected from each experimental group were processed into single-cell suspensions by gently passing the tissue through 70 µm strainers (Corning). Red blood cells were removed using ACK lysis buffer (Gibco), after which the remaining lymphocytes were washed with DPBS (Sigma). Cells were plated in 24-well culture plates at a density of 1 × 10^5^ cells per well in RPMI medium (Sigma) and stimulated with recombinant MEV at final concentrations of 1, 2, or 5 µg/mL. Cultures were maintained for 48–72 hours at 37°C in a humidified incubator with 5% CO_2_. Cell proliferation was assessed by counting viable cells using a hemocytometer. After 48 hours of antigen stimulation, culture supernatants were harvested, and cytokine levels (IL-4 and IFN-γ) were determined using commercial sandwich ELISA kits (R&D Systems) according to the manufacturer’s protocol. Cytokine concentrations were derived from standard curves generated using recombinant cytokine standards.

#### 2.8.4 *In vitro* growth inhibition test

The bactericidal potential of anti-MEV sera was evaluated following previously described protocols (78, 79) with minor modifications. In brief, 4 µL of *Leptospira interrogans* serovar Pomona cultures in mid-logarithmic growth phase (approximately 4 × 10^4^ cells) were combined with 8 µL of EMJH medium. To this mixture, 4 µL of heat-inactivated anti-MEV serum, hyperimmune anti-Pomona serum, or pre-immune serum were added for 30 min 56°C. All serum samples were heat-inactivated prior to use. Cultures incubated with EMJH medium alone served as the negative control. Each condition was tested in triplicate and incubated for 12 hours at 28°C. The mean number of viable leptospires, assessed by motility, was determined using dark-field microscopy. Viability (%) was calculated as the ratio of cells in serum-treated samples to those in the negative control, multiplied by 100. Data were analyzed statistically using one-way ANOVA.

### 2.9 Statistical analysis

All statistical analyses were carried out using GraphPad Prism software version 8.0 (GraphPad Software Inc., La Jolla, CA, USA). Data are expressed as mean ± standard deviation (SD) from two independent experiments, unless specified otherwise. Comparisons between groups were made using one-way or two-way ANOVA, followed by Dunnett’s *post hoc* multiple comparisons test. A p-value less than 0.05 was considered to indicate statistical significance.

## 3 Results

### 3.1 Identification of potential vaccine candidates using reverse vaccinology

**Figure 1:**
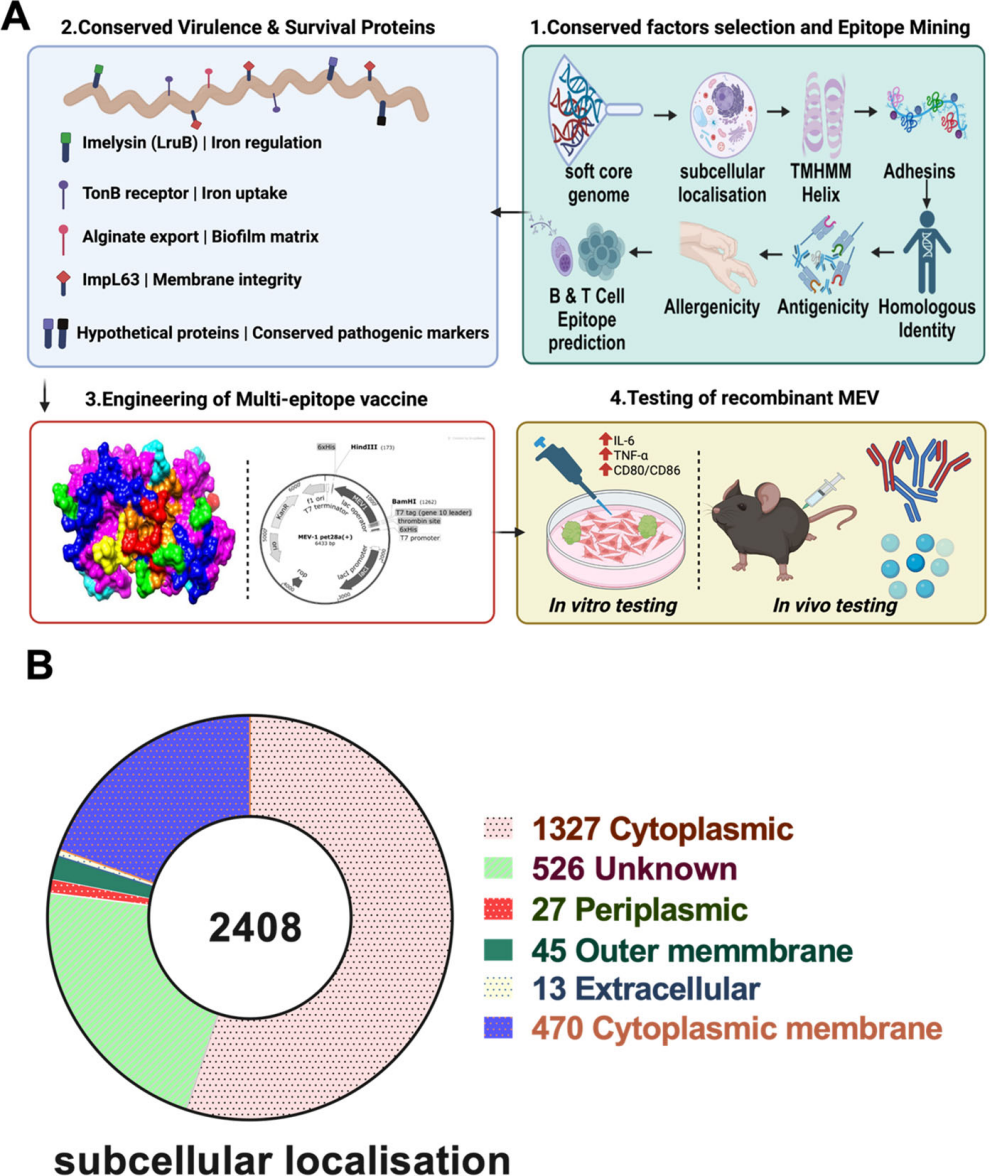
Reverse Vaccinology pipeline & Localization. **(A)** Schematic representation of reverse vaccinology pipeline and work flow designed for the study. **(B)** Sub cellular localization of soft core genome of pathogenic *Leptospira*.

In our previous study, comparative genomic analyses of sequenced *Leptospira* genomes identified 21 pathogenic species belonging to the P1 clade (37). The soft-core genome of these pathogenic *Leptospira* consists of 2,408 genes, with 1,478 conserved across all species and the remainder present in at least 95% of species. This conserved genome set served as the starting point for reverse vaccinology analysis. As illustrated in **Figure 1A**, the reverse vaccinology pipeline was employed to design a multi-epitope vaccine (MEV).

Subcellular localization analysis predicted 45 outer membrane and 13 extracellular proteins (**Figure 1B**, **Supplementary Table 3**). Of these 58 proteins, three with multiple transmembrane helices were excluded, leaving 55 proteins with one or no transmembrane helices for further analysis. Adhesin potential was evaluated, resulting in 22 proteins with credible adhesin-like characteristics based on a threshold adhesin probability of 0.51 (**Supplementary Table 4**). None of these adhesins showed homology to human proteins. Signal peptides were removed to focus antigenicity predictions on functional protein regions. Nineteen of the 22 adhesins exhibited antigenicity scores above 0.5 by VaxiJen and were considered potential vaccine candidates (**Table 1**). One outer membrane protein (WP_011671323.1) was identified as allergenic and excluded (**Supplementary Table 5**). All 18 remaining candidates demonstrated protegenicity scores exceeding 90, supporting their suitability as protective antigens (**Table 2**).

**Table 1:** Overview of characteristics of 19 adhesins identified in the research.

S. No.	Protein accession	Protein length (aa)	Localization	Localization probability	Adhesin probability	Transmembrane helices	Antigenicity
1	NP_712625.2	323	Outer Membrane	0.949	0.688	0	0.6047
2	NP_714239.2	422	Outer Membrane	0.949	0.631	1	0.7549
3	WP_011669213.1	647	Outer Membrane	0.949	0.764	1	0.6418
4	WP_011669397.1	426	Outer Membrane	0.949	0.636	0	0.6195
5	WP_011669418.1	209	Extracellular	0.964	0.749	1	0.9531
6	WP_011669449.1	252	Extracellular	0.964	0.956	0	0.9421
7	WP_011669637.1	438	Outer Membrane	1	0.822	0	0.6665
8	WP_011670051.1	547	Outer Membrane	0.949	0.651	1	0.5982
9	WP_011670465.1	610	Outer Membrane	0.949	0.808	0	0.7256
10	WP_011670651.1	235	Extracellular	0.965	0.921	0	0.6718
11	WP_011670696.1	464	Extracellular	1	0.729	0	0.7212
12	WP_011670788.1	486	Outer Membrane	0.949	0.774	0	0.6474
13	WP_011670794.1	234	Outer Membrane	0.949	0.548	1	0.8778
14	WP_011670856.1	777	Outer Membrane	1	0.544	1	0.5831
15	WP_011670925.1	472	Extracellular	0.964	0.843	0	0.5072
16	WP_011671323.1	800	Outer Membrane	0.952	0.547	0	0.5494
17	WP_011671327.1	867	Outer Membrane	0.949	0.693	1	0.6058
18	WP_011671354.1	230	Extracellular	0.965	0.941	0	0.6394
19	WP_020780498.1	234	Extracellular	0.965	0.638	0	0.8016

**Table 2:** Adhesin-like proteins have been anticipated to elicit protective immunity.

S. No	Protein accession ID	Protegenicity (%)
1	NP_712625.2	92.305931
2	NP_714239.2	93.977559
3	WP_011669213.1	99.725212
4	WP_011669397.1	94.916419
5	WP_011669418.1	93.863064
6	WP_011669449.1	90.909091
7	WP_011669637.1	98.030685
8	WP_011670051.1	97.984887
9	WP_011670465.1	99.679414
10	WP_011670651.1	91.000687
11	WP_011670696.1	99.404626
12	WP_011670788.1	94.023357
13	WP_011670794.1	93.771468
14	WP_011670856.1	98.419968
15	WP_011670925.1	90.909091
16	WP_011671327.1	99.748111
17	WP_011671354.1	90.909091
18	WP_020780498.1	92.534921

Immunological profiling involved prediction of B-cell epitopes using BCPREDs and BepiPred, yielding 181 initial epitopes, of which 149 high-confidence epitopes remained after removing low antigenicity sequences (**Supplementary Table 6**). BepiPred further refined these to 72 confident B-cell epitopes distributed across all antigens (**Supplementary Table 7**, **Table 3**). Each antigen contained at least one epitope, with WP_011671327 and WP_011670696 containing up to seven. T-cell epitope prediction identified between 10 and 94 MHC-I binding epitopes and 15 to 87 MHC-II binding epitopes per antigen. Notably, NP_714239 and WP_011671327 exhibited epitopes binding to all 27 reference MHC-I alleles, while two other antigens had epitopes binding to 19 MHC-II alleles (**Table 4**). WP_011671327 had the highest number of total T-cell epitopes (168), covering all 27 MHC-I and 19 MHC-II alleles. Promiscuous epitopes, defined as those binding to four or more MHC alleles, were identified, resulting in 69 epitopes (29 MHC-I and 40 MHC-II). Thirteen antigens contained promiscuous MHC-I epitopes, eight had promiscuous MHC-II epitopes, and seven harbored promiscuous epitopes for both classes (**Supplementary Tables 8**, **9**). For example, NP_712625 had four promiscuous MHC-I epitopes binding 13 alleles, while WP_011670465 contained ten promiscuous MHC-II epitopes. Immunogenicity scores were positive for 12 of the 18 antigens (**Supplementary Table 10**).

**Table 3:** Prediction of B cell epitopes using two tools BCPREDS and Bepipred.

S. No	Protein accession ID	BCPREDS predicted epitope	BCPREDS epitope with higher antigenicity	BCPREDS epitope overlapped with bepiPred
1	WP_020780498	5	4	1
2	WP_011671354	6	4	2
3	WP_011671327	20	15	7
4	WP_011670925	13	9	5
5	WP_011670856	17	14	6
6	WP_011670794	6	6	6
7	WP_011670788	12	9	2
8	WP_011670696	12	10	7
9	WP_011670651	6	6	1
10	WP_011670465	14	11	6
11	WP_011670051	14	10	3
12	WP_011669637	8	8	5
13	WP_011669449	6	6	4
14	WP_011669418	4	4	1
15	WP_011669397	11	8	3
16	WP_011669213	12	11	6
17	NP_714239	9	8	4
18	NP_712625	6	6	3
	Total	181	149	72

**Table 4:** Immunogenicity and the total count of anticipated epitopes and binding alleles for MHC-I and MHC-II reference alleles of eighteen possible antigens.

S. No.	Protein accession ID	Immuno-genicity score	MHC-I reference alleles		MHC-II reference alleles	
			Total predicted epitopes	Predicted binding alleles/total alleles	Total predicted epitopes	Predicted binding alleles/total alleles
1	NP_712625	1.51444	48	23/27	69	13/27
2	NP_714239	1.63616	53	24/27	79	19/27
3	WP_011669213	0.27117	62	25/27	87	16/27
4	WP_011669397	1.01257	47	24/27	55	7/27
5	WP_011669418	-0.22037	11	10/27	15	7/27
6	WP_011669449	-0.13648	13	12/27	25	4/27
7	WP_011669637	0.65048	26	22/27	40	13/27
8	WP_011670051	2.53251	61	27/27	69	15/27
9	WP_011670465	0.58537	52	25/27	59	15/27
10	WP_011670651	-0.16725	10	16/27	26	5/27
11	WP_011670696	0.61363	26	22/27	50	5/27
12	WP_011670788	1.59892	32	23/27	30	8/27
13	WP_011670794	-0.81439	14	13/27	38	12/27
14	WP_011670856	-0.94868	58	26/27	70	14/27
15	WP_011670925	1.9916	36	20/27	35	8/27
16	WP_011671327	4.4854	94	27/27	74	19/27
17	WP_011671354	0.59313	21	15/27	42	8/27
18	WP_020780498	-1.65195	18	21/27	21	5/27

Population coverage analysis demonstrated broad geographic representation of predicted MHC-I and MHC-II epitopes. Global coverage ranged from 92.31% to 100%, with WP_011671327 and NP_714239 achieving complete (100%) coverage. Coverage remained consistently high in India (89.58–99.99%), South Asia (91.04–100%), and Southwest Asia (83.32–99.92%), including Saudi Arabia (79.68–99.97%). Although greater variability was observed in Turkey (21.85–97.54%), key prioritized antigens maintained strong representation (>93%). All six proteins selected for MEV construction exhibited ≥99.86% global coverage and high regional representation across South and Southwest Asia, supporting their suitability for broad population applicability. These final selected antigens are marked with a “★” in **Supplementary Figure 2**.

### 3.2 Construction and evaluation of a novel multi-epitope vaccine construct

**Figure 2:**
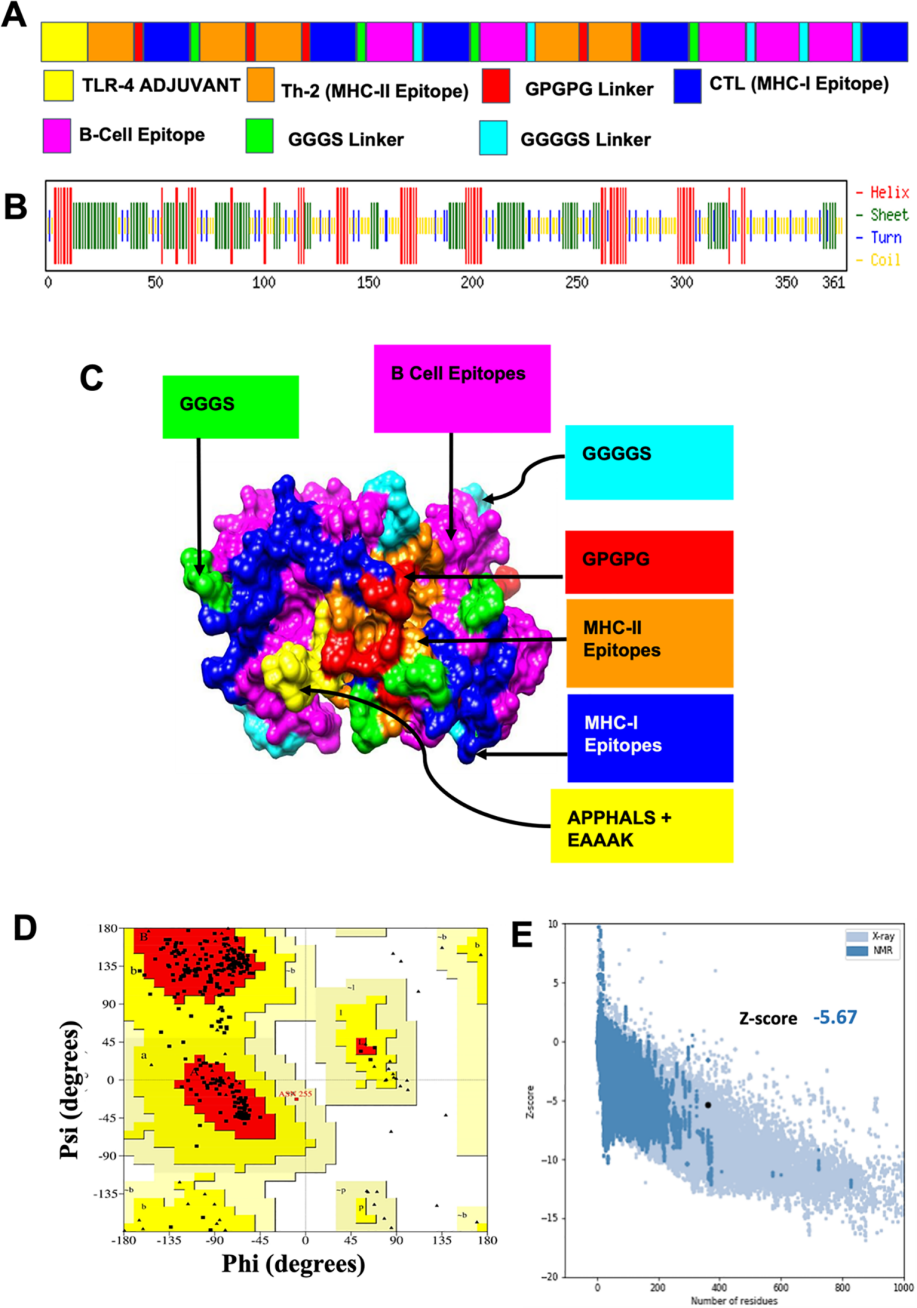
Design, construction and structural validation of multi-epitope vaccine candidate for Leptospirosis. **(A)** The predicted primary structure of the MEV with adjuvant APPHALS and linkers GPGPG, GGGS and GGGGS together with respective T cell and B cell epitopes. Structural domains and epitopes rearrangement of MEV. **(B)** Secondary structure of MEV as analyzed through CFSSP: Chou and Fasman secondary structure prediction server. **(C)** Final refined tertiary structure of MEV (surface view) visualized in UCSF Chimera; domains and epitopes are represented in different colors (APPHALS+EAAAK: Yellow, B cell epitopes: Magenta, MHC I Epitopes: Blue, MHC II Epitopes: Orange, Linkers: GPGPG: Red, GGGS: Green, GGGGS: Cyan). **(D)** Validation of the refined model with Ramachandran plot analysis showing 90.4%, 9.3% and 0% of protein residues in favored, allowed, and disallowed (outlier) regions respectively. **(E)** ProSA-web analysis with a Z-score of −5.67, indicating good model quality.

A non-redundant set of six antigens meeting all selection criteria was finalized as vaccine candidates (**Table 5**). These protein vaccine candidates (PVCs) served as the basis for developing a multivalent vaccine against leptospirosis. To enhance immune responses, a MEV was designed by combining the most immunodominant epitopes from the six PVCs. Initially, 77 epitopes including all B-cell and promiscuous T-cell epitopes were clustered into 48 unique epitopes, consisting of 39 singletons (**Supplementary Table 11**). From these, 15 epitopes were strategically selected and linked using suitable linkers to create a single MEV construct. The resulting protein comprised 361 amino acids, including the APPHALS adjuvant (**Figure 2A**).

**Table 5:** Final 6 potential vaccine candidates that qualified all the parameters against leptospirosis.

S. No	Accession ID	Immunogenicity MHC-I	No. of promiscuous Epitopes		B-Cell
			MHC-I	MHC-II	
1	NP_712625	1.51444	4	1	4
2	NP_714239	1.63616	3	7	4
3	WP_011669637	0.65048	1	4	5
4	WP_011670051	2.53251	2	5	3
5	WP_011670465	0.58537	3	10	6
6	WP_011671327	4.4854	1	8	7

Physicochemical analysis showed that the MEV construct had a molecular weight of 37.8 kDa with a theoretical isoelectric point (pI) of 4.92. The instability index was calculated at 31.14, suggesting the protein is stable under physiological conditions. The construct displayed predominantly non-polar features, with an aliphatic index of 55.46 and a GRAVY (Grand Average of Hydropathy) score of –0.384, indicating good solubility potential (**Supplementary Table 13**). Solubility prediction further supported this, with a score of 0.694, suggesting the recombinant MEV would likely remain soluble upon heterologous expression. *In silico* immunological profiling predicted the construct to be non-allergenic, anti-inflammatory, and strongly antigenic, with an antigenicity score of 1.3082, reinforcing its suitability as a subunit vaccine candidate.

Secondary structure predictions using the CFSSP server indicated that the protein is composed of 18.5% α-helices, 29.08% β-strands, and 12.4% turns (**Figure 2B**). In addition, ~6% of residues (23 amino acids) were predicted to be intrinsically disordered. Solvent accessibility mapping showed that 47% of residues were fully exposed, 24% moderately exposed, and 28% buried within the protein core.

The refined 3D structure of MEV was validated using PROCHECK, with Ramachandran plot analysis demonstrating that 90.4% of residues fell in the most favored regions and 9.3% in additionally allowed regions, with none located in disallowed regions (**Figures 2C, D**), thereby confirming structural integrity. Quality evaluation through ProSA-web produced a Z-score of –5.67, consistent with high model reliability and structural robustness (**Figure 2E**).

### 3.3 *In silico* analysis of MEV

**Figure 3:**
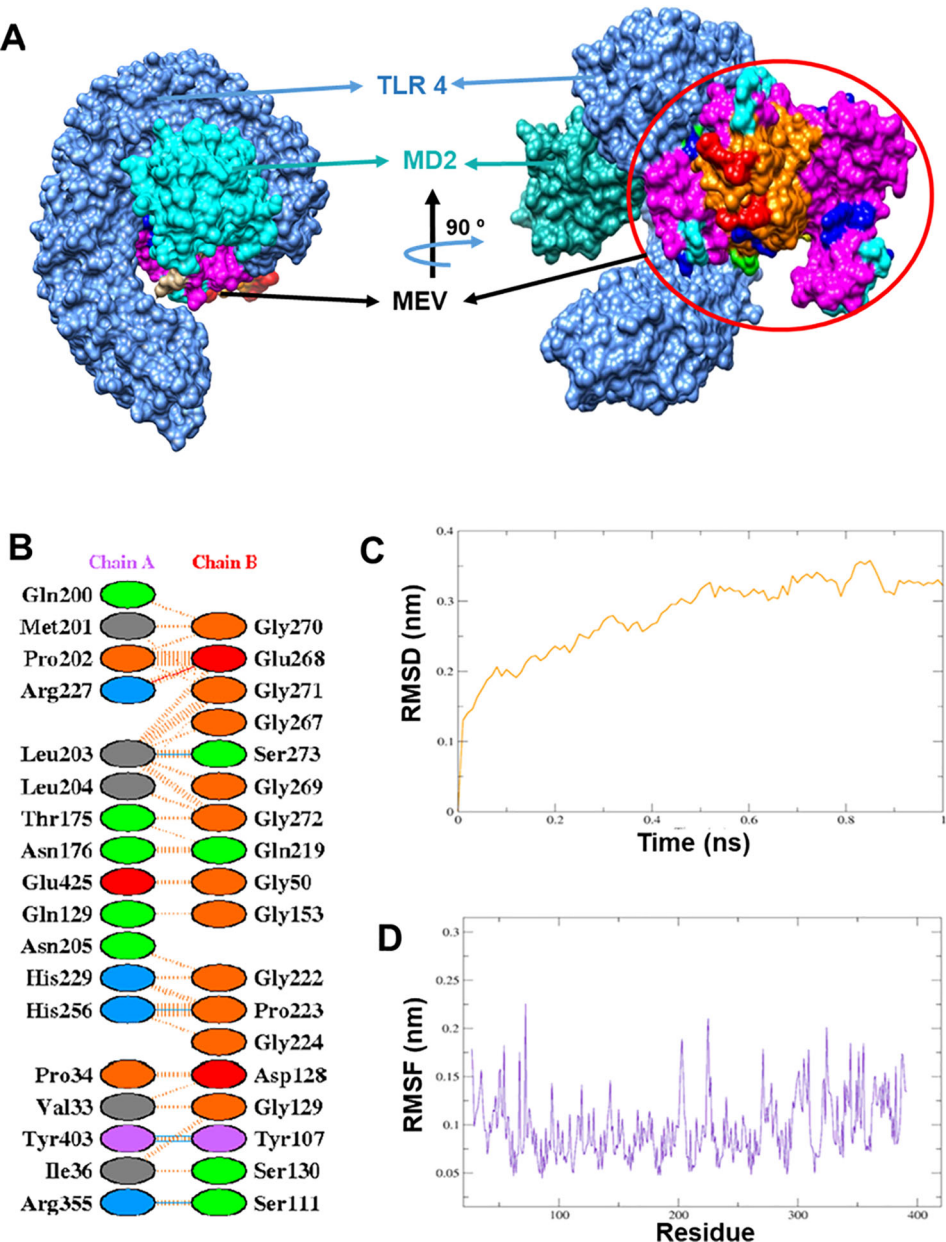
Interaction analysis between the multi-epitope vaccine (MEV) construct and the human TLR4–MD2 receptor complex. **(A)** Three-dimensional docked complex showing the MEV bound to the TLR4–MD2 complex, with TLR4 represented in corn blue and MD2 in cyan. **(B)** Two-dimensional Dimplot representation depicting hydrophobic contacts and hydrogen bonds between the MEV and TLR4, highlighting key interacting residues. **(C)** Molecular dynamics (MD) simulation analysis of the TLR4–MEV complex showing the root mean square deviation (RMSD) plot, which reflects the overall conformational dynamics and stability of the docked complex throughout the simulation period. **(D)** Root mean square fluctuation (RMSF) plot of the TLR4–MEV complex illustrating residue-wise flexibility, indicating regions of higher motion within the complex during the simulation.

The optimized MEV structure was subjected to molecular docking with the human TLR4/MD2 complex (PDB ID: 4G8A) to evaluate binding affinity and its potential to trigger an innate immune response. Twenty initial docking poses were generated, from which the top ten were refined using FireDock. Among these, the best-ranked complex displayed a global binding energy of –37.63 kcal/mol. Within this interaction, hydrogen bonds contributed –2.61 kcal/mol, while van der Waals forces showed an attractive component of –23.86 kcal/mol and a repulsive component of 10.95 kcal/mol. These values highlight a stable and favorable interaction between the MEV construct and the TLR4 receptor. The specific amino acid interactions between MEV and the TLR4/MD2 complex are depicted in **Figures 3A, B** and detailed in (**Supplementary Table 14**).

Molecular dynamics simulation analysis revealed that the root mean square deviation (RMSD) of the MEV-TLR4 complex showed initial fluctuations until 0.5 ns, after which the complex stabilized at an RMSD value of approximately 0.3 nm for the remainder of the 20 ns simulation (**Figure 3C**), indicating stable binding. The root mean square fluctuation (RMSF) plot highlighted regions of higher flexibility but maintained overall structural stability (**Figure 3D**). Radius of gyration analysis further confirmed the complex’s stability and compactness during the simulation period.

Immune response predictions using the C-ImmSim server indicated that MEV contains both B-cell and promiscuous T-cell epitopes capable of effectively triggering humoral and cellular immune responses. The primary immune response showed production of IgM and IgG antibodies (**Supplementary Figure 3A**), while the secondary response exhibited increased levels of IgG1, IgG2, IgM, and IgG+IgM, alongside significant memory B-cell development (**Supplementary Figure 3B**). Additionally, elevated IFN-γ levels were observed, reflecting Th1 cell activation. The T-helper (TH) cell population peaked during secondary and tertiary immune responses before gradually declining (**Supplementary Figure 3C**). These findings support that the MEV construct is a promising subunit vaccine candidate with strong immunogenic potential against leptospirosis.

### 3.4 Immune reactivity of purified MEV

The MEV construct (~38 kDa) was expressed and purified from *E. coli*, as confirmed by SDS–PAGE and Western blot using an anti-His antibody (**Supplementary Figure 4**). To evaluate the immunoreactivity of the purified MEV, indirect ELISA was performed using two distinct serum sources: (i) human sera, including *Leptospira*-confirmed cases by the microscopic agglutination test (MAT-positive), sera from patients with other febrile illnesses, and sera from healthy controls; and (ii) hyperimmune sera collected from mice experimentally infected with *L. interrogans* serovar Pomona, harvested 21 days post-infection.

**Figure 4:**
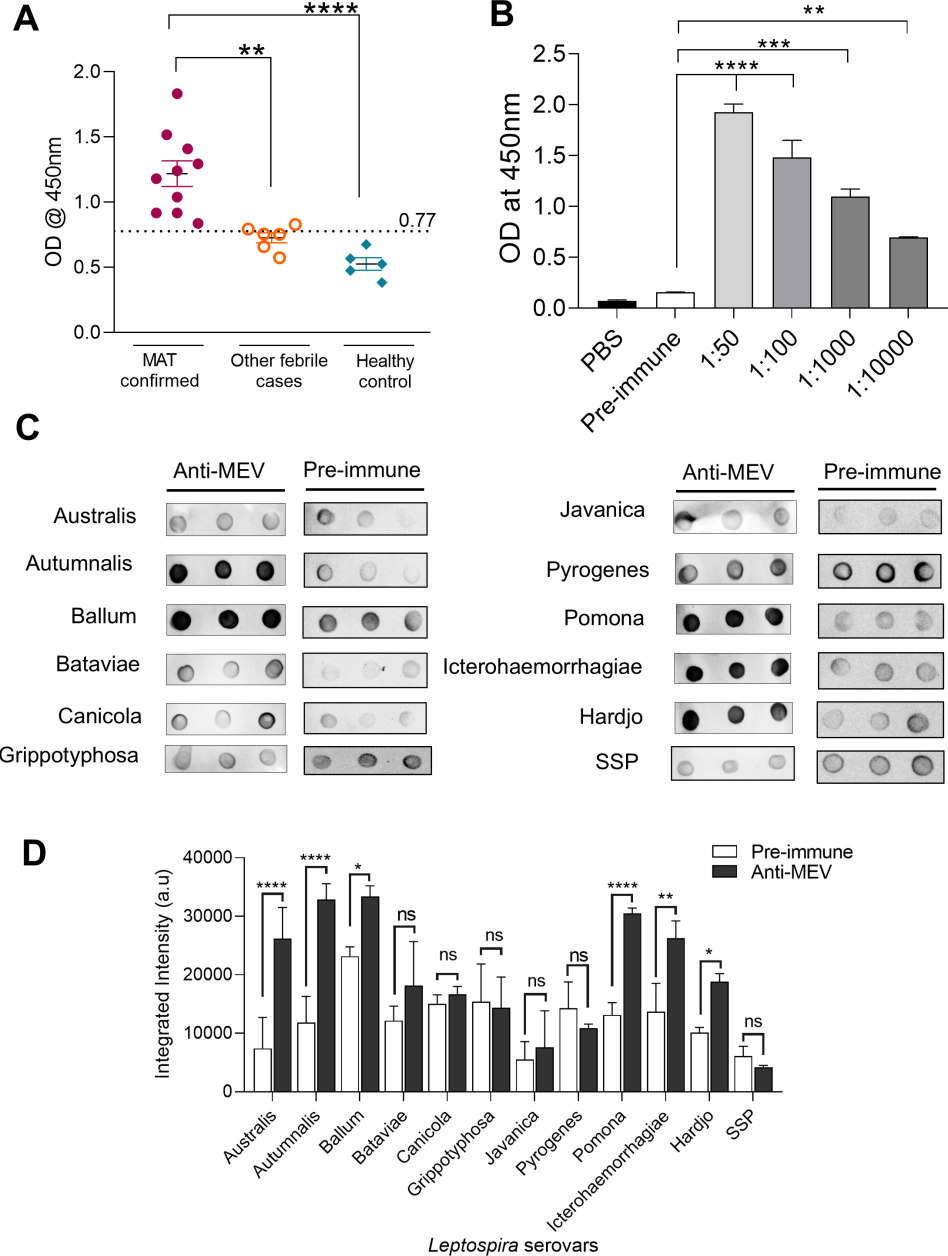
Reactivity and specificity of MEV. **(A)** Evaluation of MEV reactivity against human clinical sera, including *Leptospira*-positive MAT-confirmed sera, sera from patients with other febrile illnesses, and healthy control sera. **(B)** Reactivity of MEV against hyperimmune sera. The Y-axis represents optical density (OD) values measured at 450 nm, while the X-axis shows serial antibody dilutions of hyperimmune sera raised against *Leptospira interrogans* serovar Pomona. **(C)** Dot-blot analysis. Representative images of dot-blot assays are shown in triplicates. **(D)** Quantification of dot-blot intensity. Antibody responses elicited by MEV were assessed against different *Leptospira* serovars. The Y-axis represents integrated intensity (arbitrary units), and the X-axis denotes the tested serovars such as *L. interrogans* serovar Australis, *L. interrogans* serovar Autumnalis, *L. borgpetersenii* serovar Ballum, *L. borgpetersenii* serovar Bataviae, *L. interrogans* serovar Canicola, *L. krischneri* serovar Grippotyphosa, *L. borgpetersenii* serovar Javanica, *L. interrogans* serovar Pyrogenes, *L. interrogans* serovar Pomona, *L. interrogans* serovar Icterohaemorrhagiae, and *L. interrogans* serovar Hardjo and *L. biflexa* serovar SSP. Statistical significance is indicated as follows: *P < 0.1, **P < 0.01, ***P < 0.001, ****P < 0.0001; ns = not significant.

MEV exhibited a strong and consistent reactivity with all MAT-confirmed leptospirosis patient sera (n = 10), while demonstrating only marginal recognition of sera from other febrile illnesses (n = 6). In contrast, sera from healthy controls (n = 5) displayed negligible binding, and the cutoff threshold for positivity was determined as the mean + 2 standard deviations (SD) of absorbance values from healthy individuals (dotted line at 0.77 OD; **Figure 4A**). These results indicate that MEV possesses diagnostic relevance by effectively discriminating leptospiral infections from unrelated febrile conditions.

Reactivity analysis with hyperimmune sera raised against *L. interrogans* serovar Pomona further confirmed the immunogenicity of the MEV construct. High titers of anti-MEV antibodies were detected, with clear dose-dependent binding observed across serum dilutions (**Figure 4B**). These findings strongly suggest that MEV epitopes are expressed during natural infection and that the elicited antibodies are capable of recognizing corresponding native leptospiral antigens *in vivo*.

To determine the breadth and specificity of the immune response, dot blot assays were performed using whole-cell lysates from a panel of clinically relevant *Leptospira* serogroups (**Supplementary Table 1**). Antibodies raised against MEV demonstrated strong recognition of *L. interrogans* serovar Autumnalis, *L. borgpetersenii* serovar Ballum, *L. interrogans* serovar Pyrogenes, *L. interrogans* serovar Pomona, *L. interrogans* serovar Icterohaemorrhagiae, and *L. interrogans* serovar Hardjo, while showing comparatively weaker but detectable reactivity toward *L. interrogans* serovar Australis, *L. borgpetersenii* serovar Bataviae, *L. interrogans* serovar Canicola, *L. krischneri* serovar Grippotyphosa, *L. borgpetersenii* serovar Javanica, and the non-pathogenic *L. biflexa* serovar SSP (**Figures 4C, D**). In contrast, pre-immune sera exhibited minimal reactivity against most of the tested serovars, with comparatively higher reactivity observed only towards *L. krischneri* serovar Grippotyphosa and *L. interrogans* serovar Pyrogenes. This baseline reactivity may be attributable to prior environmental exposure of the animals or to the presence of naturally occurring cross-reactive antibodies. Nonetheless, the overall binding pattern predominantly reflects antibodies induced following MEV immunization. The observed differential reactivity suggests that MEV captures conserved immunodominant epitopes across multiple pathogenic serogroups while maintaining selectivity against unrelated bacterial proteins. Complementary MAT analysis corroborated these findings by revealing consistent serogroup-specific agglutination patterns mediated by anti-MEV sera (**Supplementary Figure 5**).

Taken together, these results demonstrate that MEV is specifically recognized by sera from both naturally infected humans and experimentally infected animals. Furthermore, MEV-induced antibodies exhibit broad recognition of native leptospiral antigens spanning multiple clinically important serogroups, highlighting the potential of MEV as a cross-protective immunogen with both diagnostic and vaccine applicability.

### 3.5 Innate immune activity of MEV

**Figure 5:**
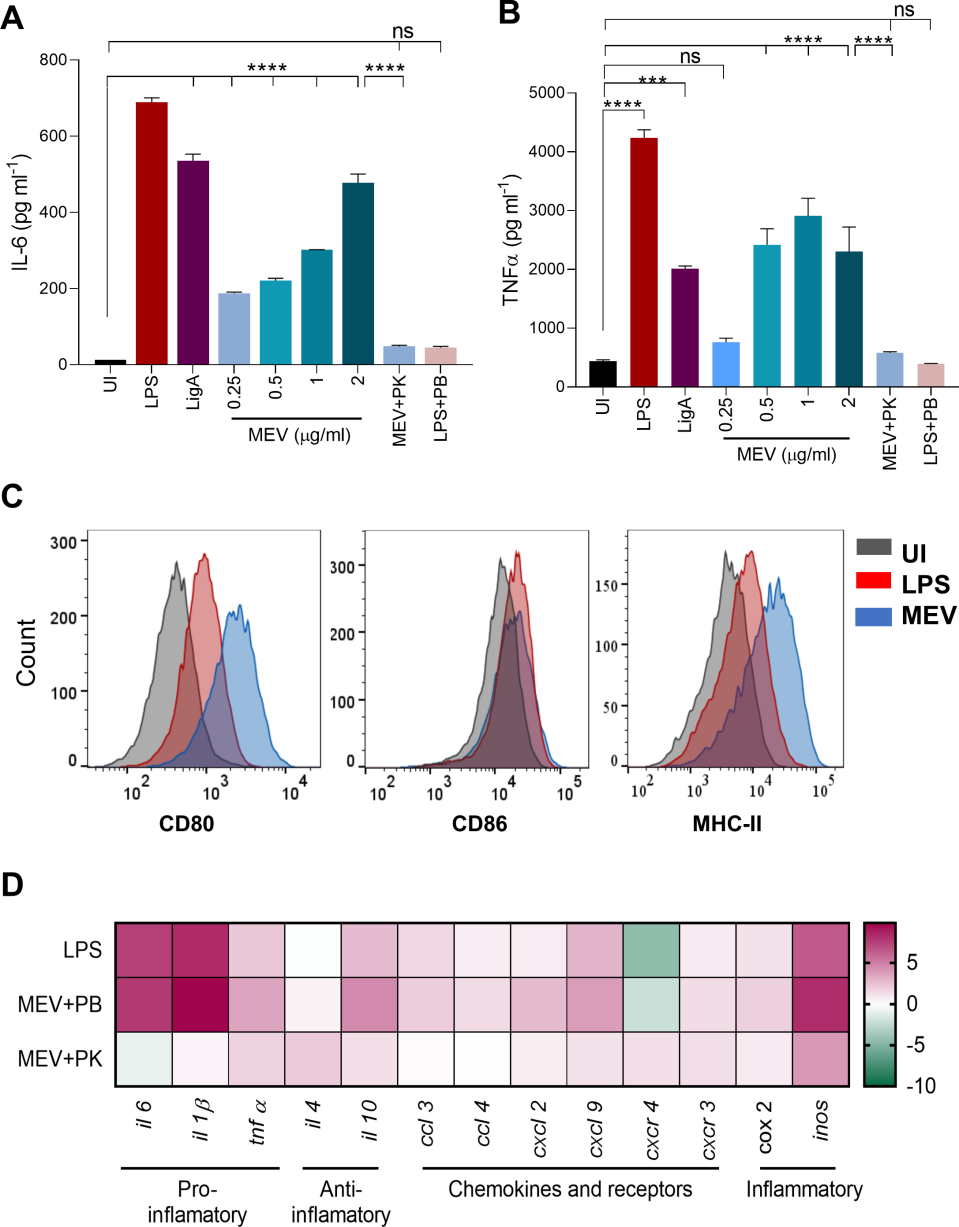
Innate immune response induced by the multiepitope vaccine (MEV). **(A, B)** Cytokine secretion in mouse macrophages following MEV stimulation. RAW 264.7 cells were treated with *Escherichia coli* LPS (500 ng/mL), MEV (0.25–2 μg/mL), LigA (2 μg/mL) Polymyxin B (PB, 10 μg/mg), or Proteinase K (PK, 5 μg/mg) as indicated. Recombinant LigA was used as a protein control for comparison of innate immune activation. Cytokine concentrations of IL-6 and TNF-α (Y-axis; pg/mL) were quantified by ELISA, and treatment groups are shown on the X-axis. **(C)** MEV-induced expression of co-stimulatory and antigen-presenting markers. RAW 264.7 cells were stimulated as above, stained with fluorochrome-conjugated anti-CD80, anti-CD86, and anti-MHC-II antibodies, and analyzed by flow cytometry. UI = uninduced cells; LPS = *E. coli* LPS (500 ng/mL); MEV = 1 μg/mL. **(D)** MEV-induced gene expression profiles in RAW 264.7 cells. After 24 h stimulation, total RNA was extracted, reverse-transcribed into cDNA, and target gene expression was quantified by RT-qPCR. Data are shown as Log2fold change relative to unstimulated controls and normalized to GAPDH. Results are expressed as mean ± SD. Statistical significance was determined using one-way ANOVA followed by Dunnett’s *post hoc* test: ***p < 0.001, ****p < 0.0001, ns, not significant.

To compare the innate immune activation potential of MEV with a known leptospiral protective antigen, RAW 264.7 macrophages were stimulated with MEV or recombinant LigA. MEV stimulation resulted in a robust pro-inflammatory cytokine response, characterized by dose-dependent production of IL-6 and TNF-α (**Figures 5A, B**). To confirm that this activity was not due to endotoxin contamination, MEV preparations were pre-treated with Polymyxin B (PB) prior to cell stimulation. As a control, LPS (500 ng/ml) pre-incubated with PB failed to induce cytokine production, validating the efficacy of PB in neutralizing LPS activity. Importantly, PB treatment did not abolish MEV-induced cytokine production, whereas digestion with Proteinase K completely abrogated it, indicating that the stimulatory effect was protein-specific and not due to LPS contamination.

Flow cytometry analysis further demonstrated that MEV significantly upregulated the surface expression of co-stimulatory molecules (CD80, CD86) and the antigen-presentation marker MHC-II on macrophages (**Figure 5C**), consistent with an activated phenotype.

To further investigate the immunomodulatory properties of MEV, we profiled the transcriptional response of innate immune-related genes using RT-qPCR. MEV stimulation resulted in robust upregulation of chemokines (CCL3, CCL4, CXCL2, CXCL9) and their receptors (CXCR4, CXCR3), as well as pro-inflammatory cytokines such as IL-6, TNF-α, and IL-1β. Moreover, key inflammatory mediators, including inducible nitric oxide synthase (iNOS) and cyclooxygenase-2 (COX-2), were significantly induced following treatment (**Figure 5D**). The breadth of this transcriptional response closely resembled that triggered by LPS, highlighting MEV’s ability to activate macrophages effectively. Collectively, these results indicate that MEV not only stimulates innate immune signaling but also drives a broad inflammatory gene expression program, supporting its potential as a strong immunostimulatory vaccine candidate.

### 3.6 Immunogenicity of MEV in mice

**Figure 6:**
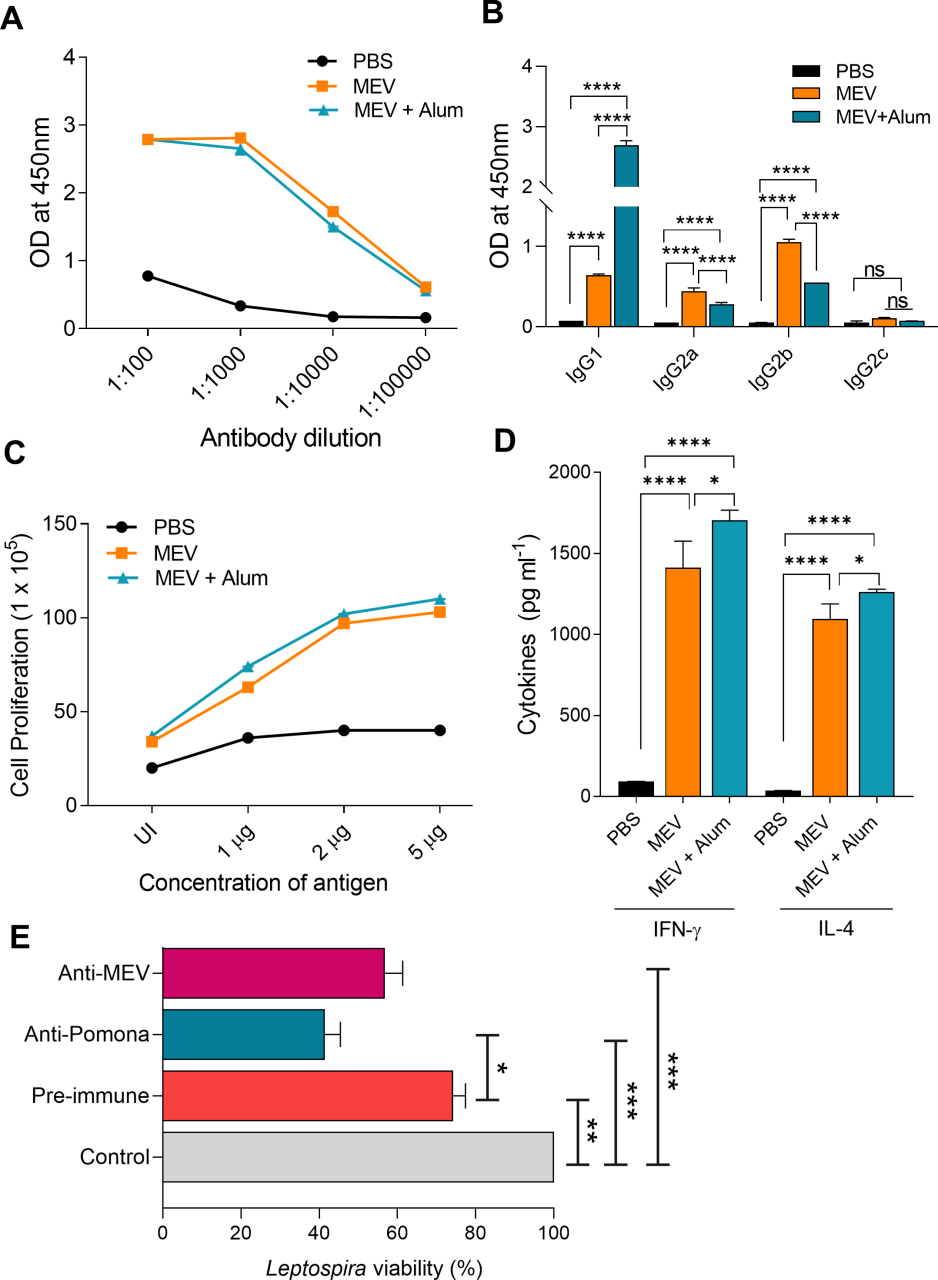
Humoral immune responses and bactericidal activity of anti-MEV antibodies. **(A)** MEV-specific total IgG titers in mouse serum at day 28 post-immunization, measured by ELISA across serial antibody dilutions. **(B)** Serum IgG isotype profiles at day 28, determined by ELISA. **(C)** Lymphocyte proliferation assay. Splenocytes from immunized and control mice were stimulated with MEV at 1, 2, or 5 μg/mL, and cell proliferation was assessed after 48 (h) **(D)** Cytokine secretion profile. IL-4 and IFN-γ levels in the culture supernatants of MEV-stimulated splenocytes were quantified by ELISA. **(E)** Bactericidal activity of anti-MEV antibodies against *Leptospira interrogans* serovar Pomona. Groups included: Control (healthy mouse sera), pre-immune sera, hyperimmune sera (day 21 post-infection with *L. interrogans* Pomona), and anti-MEV sera. The Y-axis represents percent viability of bacteria, calculated from the number of motile leptospires per microscopic field relative to the control group (only EMJH medium), assessed by dark-field microscopy. All experiments were performed in two independent biological replicates with four animals per group (n = 4). Data are presented as mean ± SD. Statistical significance was determined by one-way ANOVA followed by Dunnett’s *post hoc* test. Significance is indicated as follows: *P < 0.1, **P < 0.01, ***P < 0.001, ****P < 0.0001; ns = not significant.

To assess the immunogenicity of MEV and characterize the MEV-specific humoral and cellular immune responses, BALB/c mice were immunized subcutaneously with MEV alone or in combination with the adjuvant Alum. Mice immunized with MEV alone exhibited a robust antibody response by day 28 post-immunization (**Figure 6A**). MEV induced both Th1-associated antibody subclasses (IgG2a and IgG2b) and the Th2-associated subclass (IgG1), with negligible reactivity to IgG2c, consistent with the fact that BALB/c mice do not express the IgG2c subclass (**Figure 6B**).

T-cell response analysis revealed that lymphocytes from MEV-immunized mice showed significant proliferation in a dose-dependent manner and produced notable levels of IL-4 (a Th2 cytokine) and IFN-γ (a Th1 cytokine), indicating a mixed Th1/Th2 response (**Figures 6B–D**). Although the addition of Alum did not significantly increase antibody titers against MEV, lymphocytes from Alum-MEV–immunized mice produced higher levels of both IL-4 and IFN-γ compared to the MEV-alone group (**Figures 6C, D**).

Given these robust B- and T-cell responses, we next evaluated whether MEV-induced antibodies could mediate functional antibacterial activity using an *in vitro* growth inhibition test (IGIT). In serum bactericidal assays against mid-log *L. interrogans* serovar Pomona, pre-immune sera showed limited killing (~75% viability), hyperimmune anti-Pomona sera reduced viability to ~42%, and anti-MEV sera reduced viability to ~57% (**Figure 6E**). These findings indicate that antibodies recognizing MEV epitopes can contribute to bactericidal activity against *L. interrogans* serovar Pomona.

## 4 Discussion

Developing a universal vaccine against leptospirosis that elicits long-term, cross-protective immunity has remained a major challenge (14, 80, 81). Current killed vaccines provide only short-lived, serovar-specific protection and are associated with toxicity due to their lipopolysaccharide (LPS) content a T-independent antigen (82). Consequently, vaccine research has shifted toward surface proteins, which are T-dependent antigens, as potential subunit vaccine candidates. These antigens can induce long-lasting protective immunity, including memory responses (74, 82, 83). However, despite the identification of several target proteins, most candidates have offered only partial protection and have failed to prevent renal colonization an essential requirement for sterilizing immunity (14, 84, 85). Therefore, next-generation vaccine development must focus on identifying conserved, highly immunogenic multicomponent antigens capable of inducing broad, cross-protective responses.

Recent immunoinformatics-driven vaccine studies have similarly emphasized the importance of stringent antigenicity thresholds in candidate prioritization. Studies designing multi-epitope vaccines against bacterial and viral pathogens reported that selecting epitopes with VaxiJen scores >0.5 significantly improves predicted immunogenicity and structural compatibility with immune receptors (86). Our findings are consistent with these reports, supporting the robustness of antigenicity-based filtering in reverse vaccinology pipelines.

In this study, we employed a comprehensive immuno-informatics and reverse vaccinology approach to identify vaccine targets from the soft-core genome of pathogenic *Leptospira* species. Proteins were shortlisted based on stringent selection filters, including non-homology to host proteins, absence of allergenic potential, strong antigenicity, adhesive properties, and association with virulence. The prioritized proteins harbored multiple high-affinity MHC-I and MHC-II binding epitopes, as well as linear B-cell epitopes. In total, 69 promiscuous epitopes were identified, comprising 29 that interact with reference MHC-I alleles and 40 that recognize reference MHC-II alleles. Together, these epitopes provide an estimated population coverage of more than 90% globally and nearly complete coverage within South Asia, underscoring their broad immunological relevance. The use of multiple prediction tools for B-cell epitope identification reduces individual algorithm bias and improves reliability. Recent multi-epitope vaccine studies have also adopted consensus approaches combining linear and conformational prediction methods to enhance specificity and immunogenic accuracy (87). Our consensus-based strategy strengthens confidence in the predicted B-cell epitopes and aligns with current best practices in epitope-based vaccine design.

To maximize immunogenic potential, epitopes were selected to bind across a wide range of HLA molecules, since recognition of peptide–MHC-I (pMHC) complexes is known to be critical for initiating strong and durable immune responses, thereby extending the benefit beyond epitope diversity alone (88, 89). For T-cell epitope prediction, recent studies have prioritized promiscuous MHC-binding epitopes with broad HLA coverage and strong binding affinities, often validated through molecular docking and immune simulation analyses (32, 87) Similar to these competitive studies, our selected epitopes demonstrated favorable population coverage and structural interaction profiles, reinforcing their potential to elicit effective cellular immune responses.

Six proteins were prioritized as potential vaccine candidates. Two (NP_714239, NP_712625) belong to the outer membrane protein family, involved in adhesion, invasion, immune evasion, and virulence. WP_011671327, a TonB-dependent receptor, may induce protective immunity by blocking bacterial iron acquisition (90, 91). WP_011670465, an alginate export family protein, contributes to biofilm formation and virulence (92). WP_011669637 (Imelysin/LruB) participates in iron acquisition and exhibits proteolytic activities (93). WP_011670051 is a transmembrane protein (Impl63) of unknown function found in pathogenic *Leptospira*.

For MEV assembly, we identified the most immunogenic epitopes from these proteins, assuring the inclusion of at least one MHC-I, one MHC-II, and one B-cell epitope from each, incorporating overlapping sequences. Glycine-rich linkers were incorporated to optimize flexibility, folding, stability and immunogenicity (94). Physicochemical analysis showed an appropriate molecular weight for expression, an isoelectric point (pI) of 4.92, hydrophilic character, a high codon adaptation index (CAI = 0.98) and GC content of 33.06%, all consistent with good expression and solubility. Ramachandran plot based structural validation confirmed the stereochemical reliability of the model (95).

To enhance immunogenicity, we incorporated the TLR4 agonist peptide APPHALS at the N-terminus via an EAAAK linker. TLR4 activation is known to boost protective immunity in various models (96). Docking results indicated stable binding between MEV and the human TLR4-MD2 complex (binding energy –37.63 kcal/mol), supporting the potential for strong innate immune activation (97). In addition, *in-silico* immunogenicity analyses predicted that the construct would elicit robust immune responses, with epitopes positioned for efficient recognition by antibodies, B cells, and antigen-presenting cells. The simulations also indicated the potential for generating multiple antibody isotypes (IgM, IgG, IgG1, IgG2b, IgG2c) and elevated cytokines (IL-10, IL-2, IFN-γ), further supporting the biological feasibility of the MEV design.

Experimental validation confirmed the *in silico* predictions that, the MEV exhibited high expression, solubility, and purity, and also reacted strongly with both human clinical and hyperimmune serum samples, consistent with earlier reports on recombinant multi-epitope *Leptospira* proteins (r-LMP) and chimeric vaccines (rChi) (34, 98). To confirm antigenic reactivity, we performed a microscopic agglutination test (MAT) to assess the ability of anti-MEV antibodies to agglutinate multiple *Leptospira* serovars prevalent in the Indian subcontinent. Apart from its role in serodiagnosis, MAT can determine the agglutinating capacity of antibodies (99, 100). Anti-MEV sera agglutinated most of the tested serovars, suggesting that the designed MEV includes epitopes from antigens conserved across diverse serovars, leading to the generation of cross-agglutinating antibodies.

Since MEV was designed using epitopes from surface-exposed and secretory proteins with known immunomodulatory roles, we assessed its effect on innate immune activation, as innate responses are critical for initiating antigen-specific adaptive immunity. MEV stimulation led to pronounced macrophage activation and maturation, evidenced by elevated proinflammatory cytokine production, increased expression of co-stimulatory molecules, and modulation of innate immunity-related genes. These findings are consistent with earlier reports showing that protective *Leptospira* antigens can activate innate immune cells (74, 101). While the precise TLR pathways remain to be confirmed, activation via both TLR2 and TLR4 is likely, given that MEV incorporates a TLR4 agonist and that several *Leptospira* proteins have been reported to engage these receptors (73, 74).

To experimentally validate the *in silico* predicted immunogenicity of MEV, we tested it in a murine model. MEV elicited a robust antibody response, with high titers of total IgG and both IgG1 and IgG2a isotypes. Notably, the inclusion of alum did not markedly enhance antibody production, indicating that MEV is intrinsically immunogenic. However, alum significantly increased IL-4 and IFN-γ secretion, suggesting an adjuvant effect on cytokine profiles. Consistent with previous reports highlighting the importance of CD4^+^ T cells and IFN-γ in protective immunity against leptospirosis (102, 103), these findings support MEV’s potential to stimulate both humoral and cellular arms of the immune system. Although protective efficacy could not be assessed in a hamster challenge model, the neutralizing capacity of MEV-induced antibodies was confirmed using an *in vitro* growth inhibition test (IGIT), a standard approach for evaluating *Leptospira* vaccine potency (104). Serum from MEV-immunized mice inhibited the growth of *L. interrogans* serovar Pomona, aligning with prior studies employing growth inhibition assays to detect cross-neutralizing antibodies (35). Since antibodies contribute to bacterial clearance via complement-mediated lysis and opsonin-dependent phagocytosis (105, 106), the neutralizing antibodies against MEV revealed growth inhibition of the leptospires suggesting that MEV may confer cross-protective immunity, warranting further *in vivo* validation.

Collectively, our integrative immunoinformatic pipeline, supported by stringent antigenicity filtering, consensus B-cell epitope prediction, promiscuous T-cell epitope selection, broad HLA population coverage analysis, and experimental validation, strengthens the translational relevance of the proposed multi-epitope vaccine. Compared to recent reverse vaccinology studies, our framework not only aligns with emerging best practices but also extends them by integrating immunogenicity scoring and *in vitro* functional validation. These findings position the present MEV construct as a rationally designed and competitively benchmarked candidate for further preclinical evaluation against leptospirosis.

In conclusion, using comparative genomics and reverse vaccinology, we designed a novel MEV candidate that showed strong immunogenicity *in silico*, potent innate immune activation *in vitro*, and robust antibody responses *in vivo*. Sera from leptospirosis patients recognized MEV epitopes, suggesting that these regions are targeted during natural infection and supporting their relevance for vaccine development. Anti-MEV antibodies from immunized mice agglutinated *Leptospira* in MAT and inhibited bacterial growth *in vitro*, indicating the potential of MEV-induced antibodies to target different strains. These findings, together with the conserved nature of the selected epitopes, support MEV as a promising next-generation multi-epitope vaccine candidate for leptospirosis. The present study was primarily focused on the rational design and immunogenicity evaluation of the MEV construct. Due to constraints related to the availability of a suitable virulent challenge strain, protective efficacy studies in a susceptible animal model could not be performed at this stage. We are currently planning follow-up studies to evaluate the protective efficacy of the MEV candidate in a susceptible hamster model of leptospirosis. Further studies assessing long-term immunity, memory responses, and protective efficacy in challenge models will be important steps toward its preclinical and clinical evaluation for both human and veterinary use.

## Data Availability

The original contributions presented in the study are included in the article/**Supplementary Material**. Further inquiries can be directed to the corresponding author/s.

## Supplementary material

Supplementary Figure 1 Bepipred output of each Protein Antigen: Amino acid with score above threshold value are depicted as in yellow in the figure.

Supplementary Figure 2 Population coverage of 18 antigens by IEBD standalone population coverage.

Supplementary Figure 3 Immune simulation by C-ImmSim server **(A)** Both IgG and IgM reached their maxima after MEV vaccine induction. **(B)** The immunoglobulins and the immunocomplex response to antigen (MEV) inoculations (black vertical lines); specific subclasses are indicated as colored peaks, **(C)** Evolution of Th cells with course of vaccination and induction of interleukins after vaccination with MEV.

Supplementary Figure 4 **(A)***In silico* fusion cloning of the MEV gene into the pUC57 vector using EcoRI (3′ end) and HindIII (5′ end) restriction sites. **(B)** Final vaccine construct inserted into the pET28a+ expression vector at EcoRI and HindIII sites. The red region denotes the predicted MEV gene sequence, and the black circular region represents the vector backbone. **(C)** Confirmation of MEV cloning in pUC57 by restriction digestion with EcoRI and HindIII. Agarose gel electrophoresis: lane 1, DNA ladder; lane 2, EcoRI- and HindIII-digested recombinant pUC57-MEV; lane 3, uncut recombinant vector. **(D)** SDS-PAGE analysis showing purification of recombinant MEV. **(E)** Western blot analysis of purified MEV using MEV-specific antibodies.

Supplementary Figure 5 **(A)** Agglutination of 12 live *Leptospira* serovars following incubation with polyclonal antibodies raised against MEV. Agglutination patterns were visualized by dark-field microscopy. Pre-immune serum served as the negative control, and hyperimmune sera against *L. interrogans* serovar Pomona served as the positive control. **(B)** MAT titers of anti-MEV antibodies against individual leptospiral serovars: *L. interrogans* serovar Australis, *L. interrogans* serovar Autumnalis, *L. borgpetersenii* serovar Ballum, *L. borgpetersenii* serovar Bataviae, *L. interrogans* serovar Canicola, *L. krischneri* serovar Grippotyphosa, *L. borgpetersenii* serovar Javanica, *L. interrogans* serovar Pyrogenes, *L. interrogans* serovar Pomona, *L. interrogans* serovar Icterohaemorrhagiae, and *L. interrogans* serovar Hardjo and *L. biflexa* serovar SSP.
