# Supplementary material for: A multi-epitope approach for development of a universal vaccine against leptospirosis
Source: Front Immunol. 2026 Mar 20;17:1774717. doi: 10.3389/fimmu.2026.1774717 (PMC13046716; doi:10.3389/fimmu.2026.1774717)
Supplement: Supplementary Figure 1 — Bepipred output of each Protein Antigen: Amino acid with score above threshold value are depicted as in yellow in the figure. [file DataSheet1.pdf]

Sup Fig. 1

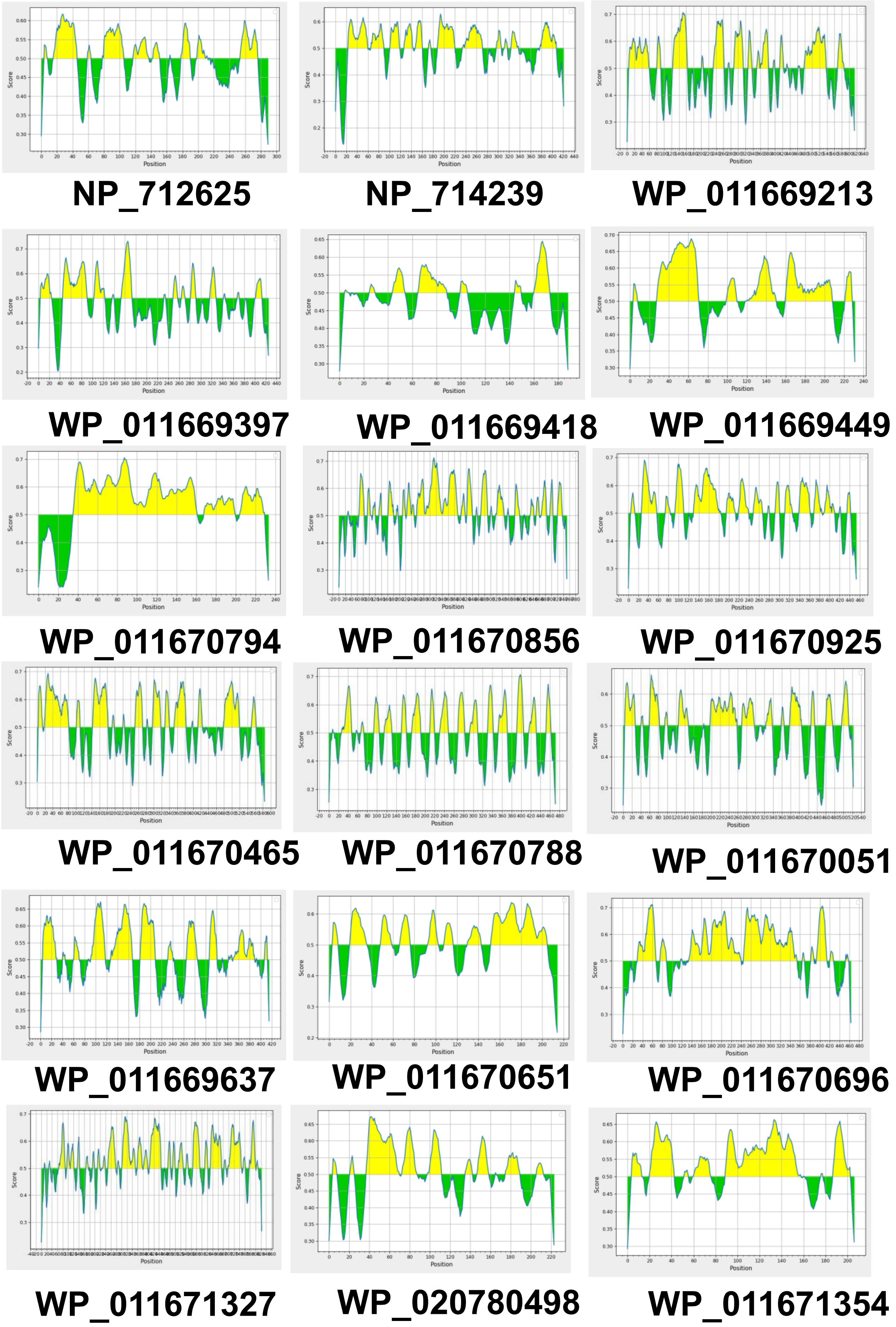
Score
Position
NP_712625
NP_714239
WP_011669213
WP_011669397
WP_011669418
WP_011669449
WP_011670794
WP_011670856
WP_011670925
WP_011670465
WP_011670788
WP_011670051
WP_011669637
WP_011670651
WP_011670696
WP_011671327
WP_020780498
WP_011671354

Sup Fig. 2

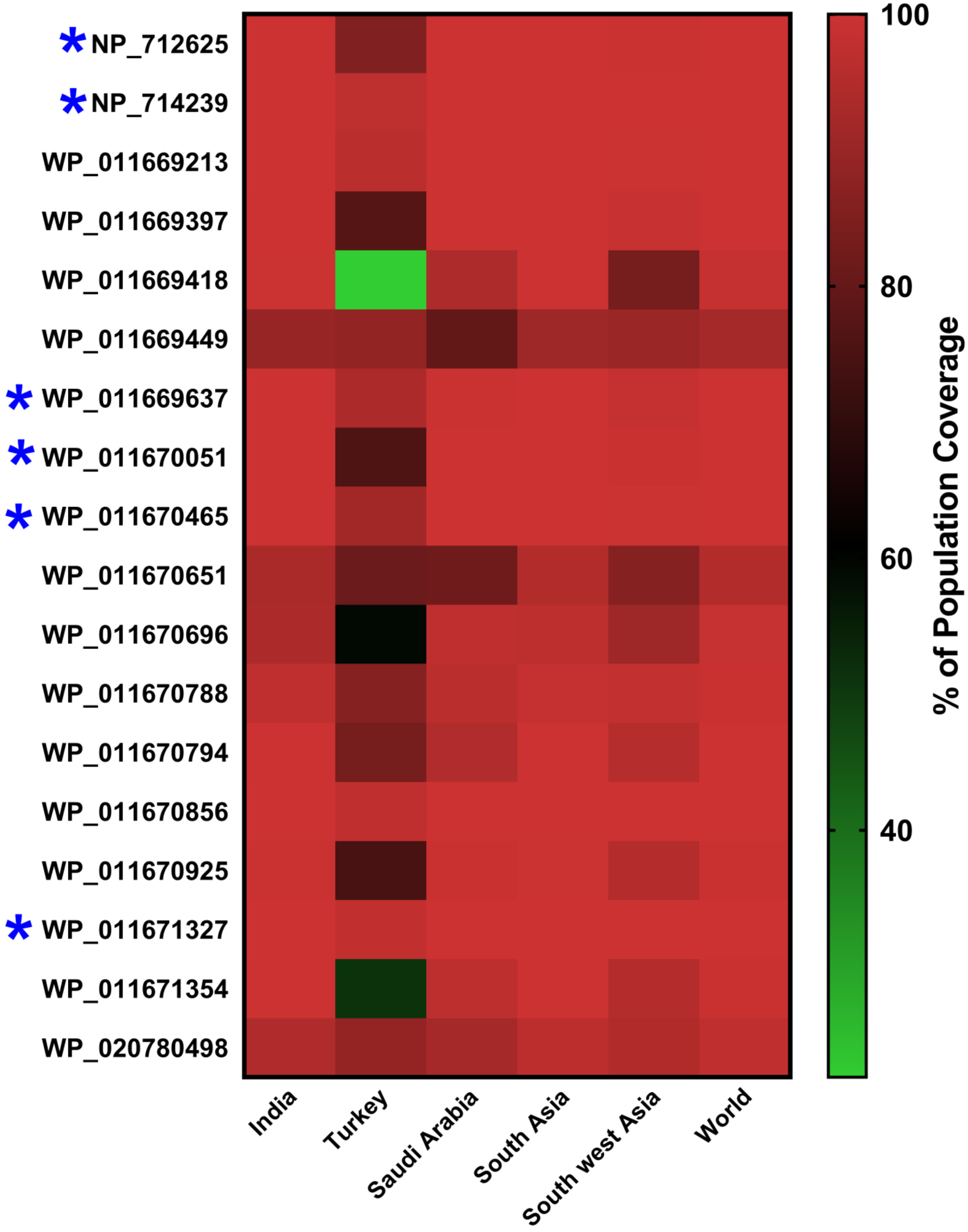

*NP_712625
*NP_714239
WP_011669213
WP_011669397
WP_011669418
WP_011669449
*WP_011669637
*WP_011670051
*WP_011670465
WP_011670651
WP_011670696
WP_011670788
WP_011670794
WP_011670856
WP_011670925
*WP_011671327
WP_011671354
WP_020780498
India
Turkey
Saudi Arabia
South Asia
South west Asia
World
100
80
60
40
% of Population Coverage

Sup Fig. 3

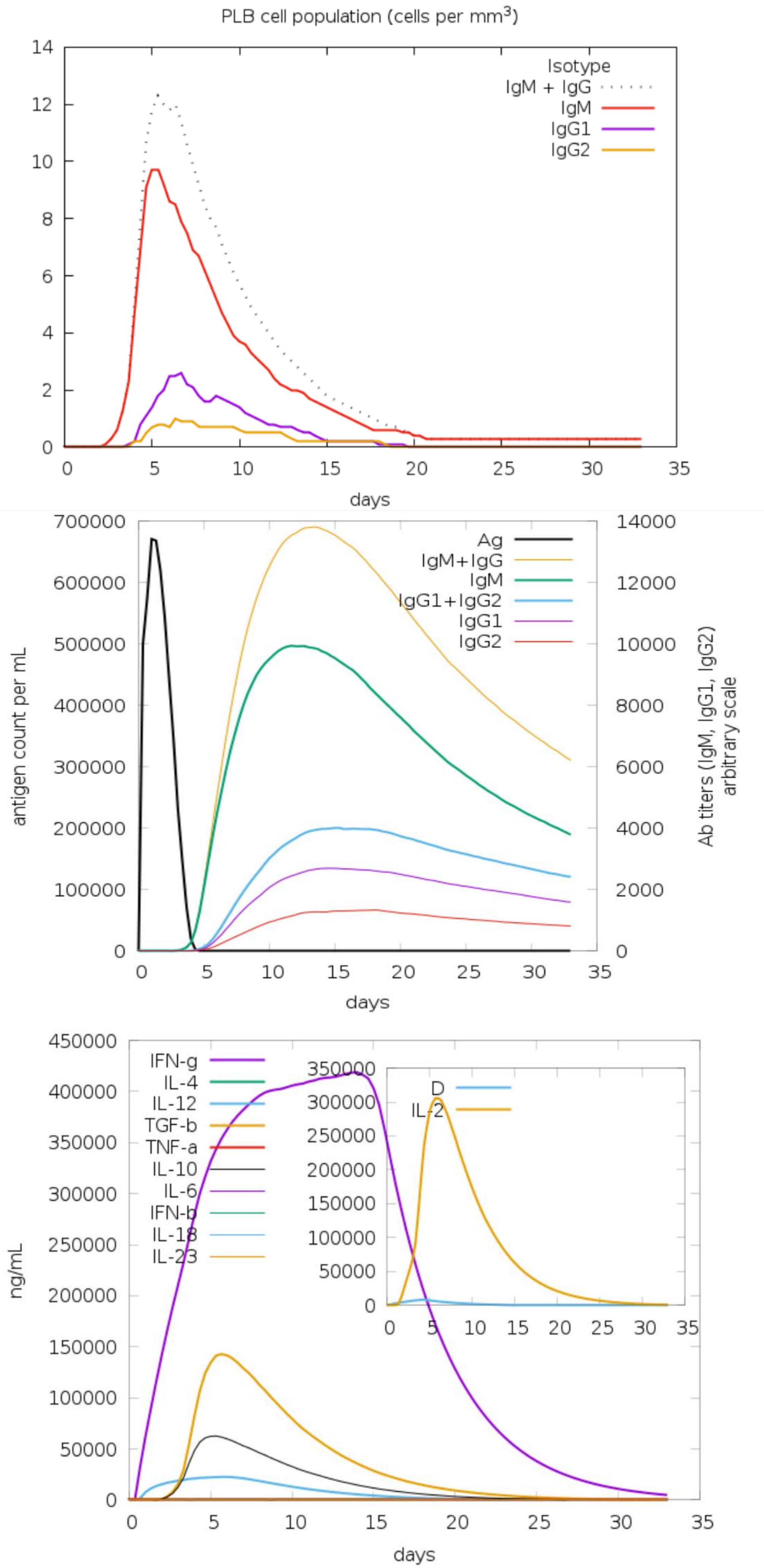
A
PLB cell population (cells per mm^3)
Isotype
IgM + IgG
IgM
IgG1
IgG2
days
B
Ag
IgM+IgG
IgM
IgG1+IgG2
IgG1
IgG2
antigen count per mL
Ab titers (IgM, IgG1, IgG2) arbitrary scale
days
C
IFN-g
IL-4
IL-12
TGF-b
TNF-a
IL-10
IL-6
IFN-b
IL-18
IL-23
D
IL-2
ng/mL
days

S. Fig. 4

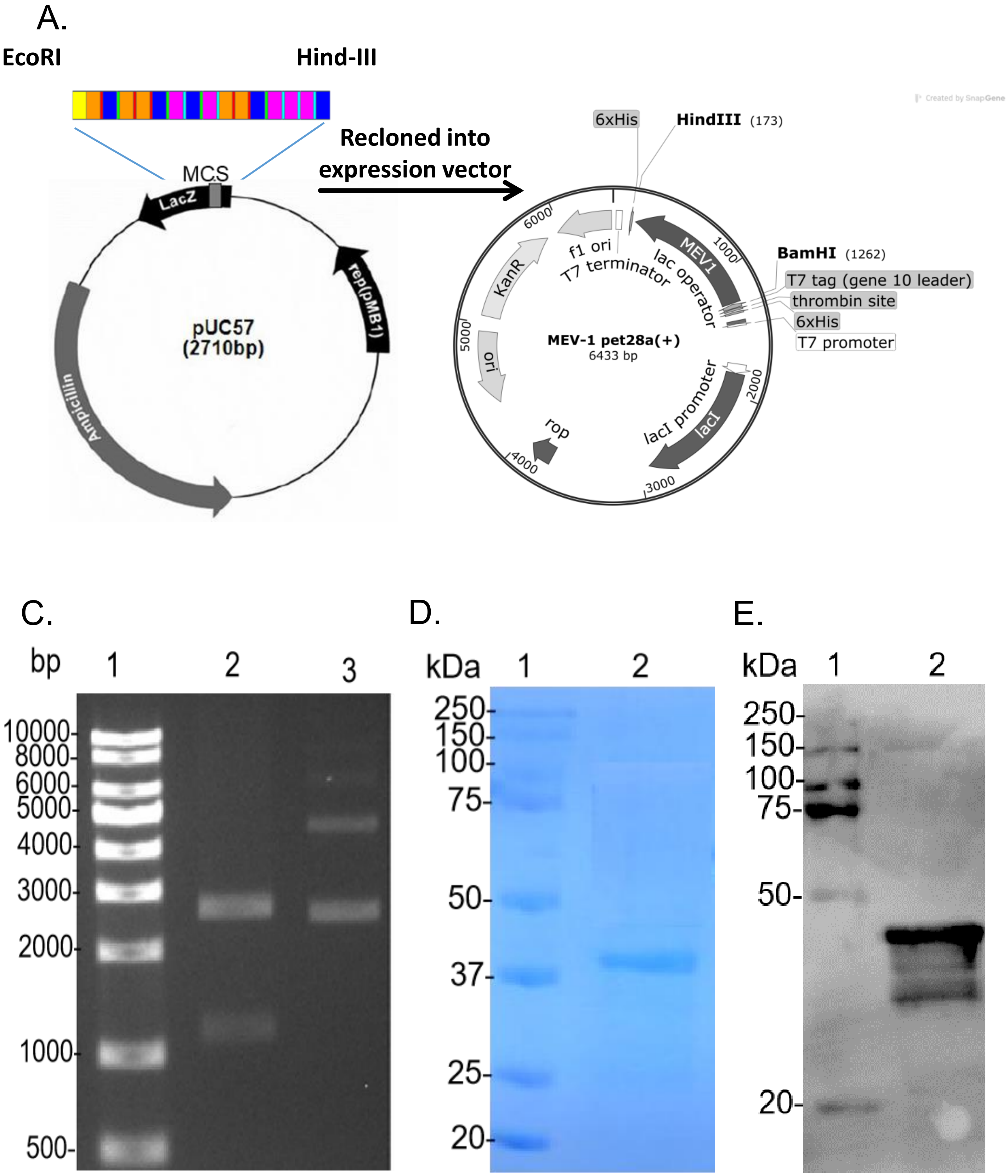
A.
EcoRI
Hind-III
MCS
LacZ
rep(pMB1)
Ampicillin
pUC57
(2710bp)
Recloned into
expression vector
Created by SnapGene
6xHis
HindIII (173)
BamHI (1262)
T7 tag (gene 10 leader)
thrombin site
6xHis
T7 promoter
MEV1
lac operator
T7 terminator
f1 ori
KanR
ori
rop
lacI promoter
lacI
MEV-1 pet28a(+)
6433 bp
1000
2000
3000
4000
5000
6000
C.
bp
1
2
3
10000
8000
6000
5000
4000
3000
2000
1000
500
D.
kDa
1
2
250
150
100
75
50
37
25
20
E.
kDa
1
2
250
150
100
75
50
20

S. Fig. 5

A.

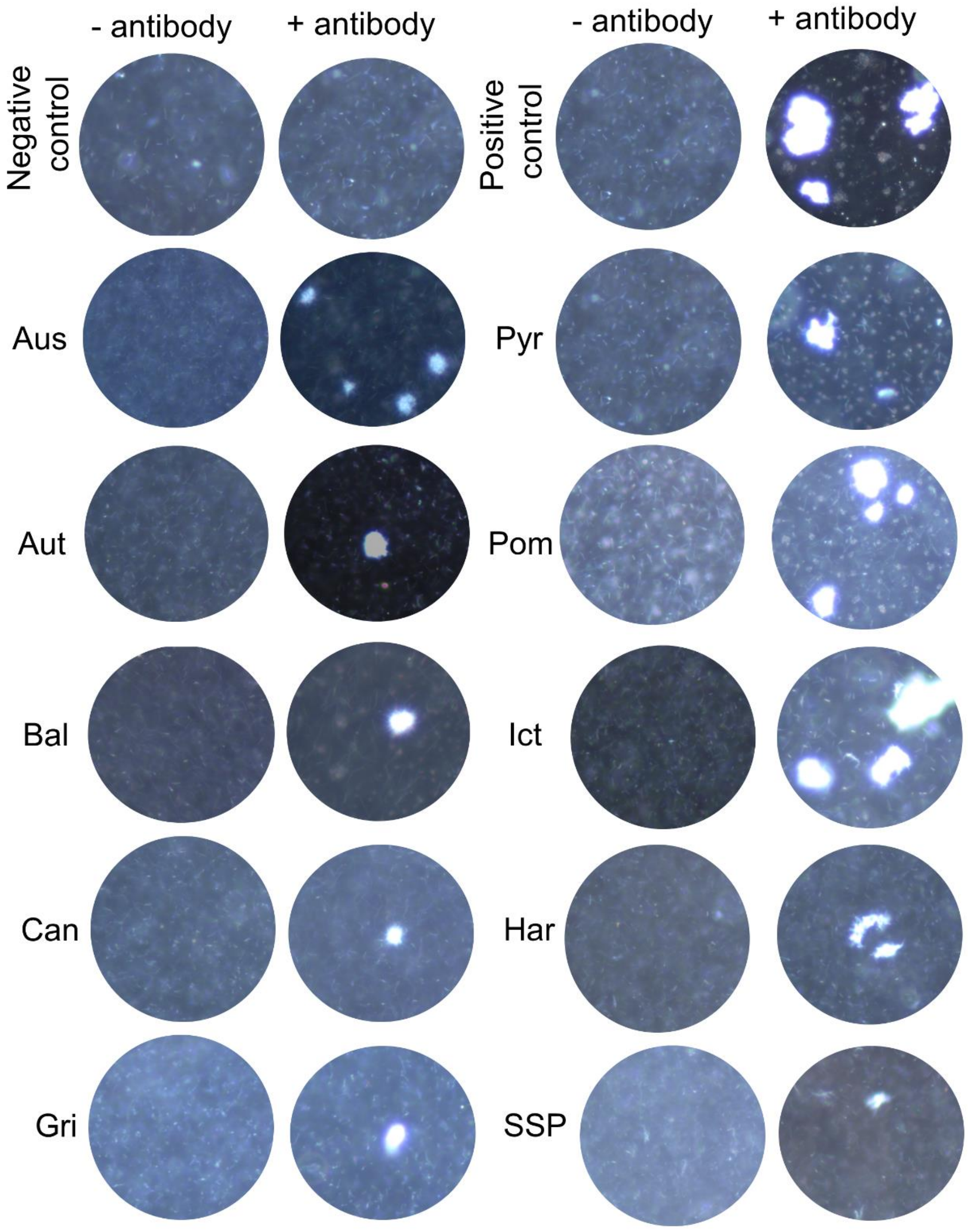

B.

Seogroup	MAT result	Seogroup	MAT result
Aus	+ve (1:40)	Jav	-ve
Aut	+ve (1:160)	Pyr	+ve (1:160)
Bal	+ve (1:160)	Pom	+ve (1:320)
Bat	-ve	Ict	+ve (1:80)
Can	+ve (1:20)	Har	+ve (1:160)
Gri	+ve (1:80)	SSP	-ve
